# Supplementary material for: Factors influencing patients on antiretroviral therapy loss to follow up: A qualitative analysis of healthcare workers perspective
Source: PLoS One. 2024 Jun 13;19(6):e0304592. doi: 10.1371/journal.pone.0304592 (PMC11175404; doi:10.1371/journal.pone.0304592)
Supplement: S1 Data — (DOCX) [file pone.0304592.s002.docx]

**Participant1**

**Speaker 1** [00:00:01] Okay. Thank you very much. My name is Ackah Isaac and I'm collecting data for this research. And this was saying we are looking at a factors influence of patient on antiretroviral therapy, lost to follow up in the Asunafo South District. So sometimes some of some people start treatment and along the way and we lose them to follow or they default. So we just want to know the factors contributing to this loss to follow up. So we are collecting data from the patients, the other stakekolders, eeh health workers so that to be able to know how to address this particular problem. So all information collected here will be used for analysis purposes will not state your name or will not associate with any opinion that you state over here. It's just for research purposes. And the findings will be used to draw up corrective measures in order to be able to address that problem or to challenge that we are currently facing with people lost to follow up. So if you agree, I will begin to ask you some few questions.

**Speaker 2** [00:01:20] I agree. All right.

**Speaker 1** [00:01:22] Thank you very much. So can you tell me your position?

**Speaker 2** [00:01:29] I'm a clinician. Prescriber.

**Speaker 1** [00:01:31] Alright. Thank you very much. So, like, I'll eem, I will be speaking both English and Twi combined. Yeah. So what do you think? Or what do you think are some of the resources that you will need as a facility in order to be able to trace defaulters?

**Speaker 2** [00:01:49] Alright. Eeeh, basically eeh, vehicles. In our area, more motorbikes to help us to trace defaulters. And secondly, we would need communication gadgets talk of mobile phones that would be able to store contacts of the patients on it. That is not really a bona fide property of anybody, but for the unit that gives care to these clients and also eeh funds to help us.

**Speaker 1** [00:02:43] As in money.

**Speaker 2** [00:02:44] Money, yes. To help us get credit. Or data to be able to help us reach these clients.

**Speaker 1** [00:02:54] Okay.

**Speaker 2** [00:02:54] I think basically that will help our work a lot in tracing defaulters.

**Speaker 1** [00:02:59] Okay. So like eeh let me see with regards to that health workers, do you think you have other competing responsibilities that makes it very difficult for them to be able to trace defaults?

**Speaker 2** [00:03:14] Yeah, there are some difficulties, eeh! Looking at the work in giving care to people living with HIV. eeh, it is, like a second duty to everybody. That is in the team of giving care. So the person's mainstream eeh work or activity. Eeeh, sometimes demanding. Such that eeh (Phone vibrated).The time to devote for caring for these people are times that the person might be using to maybe take leisure or attend his own personal stuff. So it is almost work that is sacrificial if I want to say. So the other responsibilities of the profession sometimes demand a lot of times or takes a lot of time from the eeh staff. That makes it difficult to give maximum attention to the care of the people living with HIV.

**Speaker 1** [00:04:25] So in that case, are there some incentives as a facility, are there some incentives or packages in place for people, who do you defaulting or like involve in HIV antiritroviral care?

**Speaker 2** [00:04:38] I would say no. There are no eeh incentives or anything to cushing them or motivate them to do more. Basically, there is nothing like that.

**Speaker 1** [00:04:58] Hmmm, so why is there not an incentive for them?

**Speaker 2** [00:05:02] Well, we we I personally don't know why, but if there be anything... Iet see, when people go for workshops. That is what others see as the motivation that they get. But basically, I don't see it like that as a motivation enough because basically go (Interviewee laugh) you learn many things to come and give care. You spend a great deal of time from morning to evening. You sit, get tired at the end of the day you have group assignments and group work to be done at the workshops. You basicall don't have any other time and it is more tiring going for workshops and those things. So, the person come back and basically the person is given some TNT and eeh a snack that the person is take in food (Both interviewer and interviewee laughed), that is knew as money and that is not, I mean an incentive enough. So, I believe if there is a package that motivate the person, how I mean, their movement and certain basic things. Sometimes, I mean, you come to work and then you have to buy your pen and then other writing parts to take notes and then other information. Such things if they are provided, I think or believe will give some satisfaction on the job. But basically, or as far as I know there is not any other incentive that I know that is given to staff working at the ART unit or being part of the HIV team.

**Speaker 1** [00:06:47] Well. The think you eeh, so like. When a client defaults, maybe a patient defaults in the course of their treatment. When do you think is appropriate for us to do tra... follow up or to start tracing the person?

**Speaker 2** [00:07:03] I believe it might not take so much a time. The appointment time was due and this patient didn't show up. It demands a forthright action to do the follow up. We do not have to wait any other time again. It means once you take whatever that is keeping you reminded or that is reminding you of the patient's appointment time. Maybe you are colour coding. You are missing an electronic means that reminds you of when a client is supposed to come for an appointment. And nothing reminds you that so...so and so supposed to have come for a refill or medication at this time and the person didn't show up. arrangement must be made from that point in order to get the person back to care.

**Speaker 1** [00:08:05] Care.

**Speaker 2** [00:08:05] aha... so you do not have to give a very lag time. It must be forthright. Yes.

**Speaker 1** [00:08:11] Okay. So like eeh over here as a facility. So, like when clients defaults, who actually who usually do the follow up.

**Speaker 2** [00:08:22] Okay. So among the team. We have a diverse cadre of health care professionals. So basically here, because the people that do more of the home visiting and then the outreaches at the community health nurses. We do. I mean, we leave that particular task to them. eehm in recent times the other eeh NGO. Eeeh supported nurses that are also part of us. the eeh CSOs and the are SIE personnel that are with us, that is in-person visits and then tracing. They normally do them. But on the phone call, the ART nurse and then the data officer has been doing much of them.

**Speaker 1** [00:09:23] Okay.

**Speaker 2** [00:09:24] Yes.

**Speaker 1** [00:09:25] Alright. So like eeh sometimes, too, eeh... some people also believe that people... It is difficult to trace certain clients because we don't collect enough information from these clients during the time of the other point of entry. So, what type of information do you think we should collect from clients in order to be able to effectively trace them whenever eeh they default?

**Speaker 2** [00:09:54] Okay. I think basically if there be any addition to those information that we have been using already, it would not be much. We know the house number of the person or where the person stays is important. If the person has one. It is a key information that we would need and the contact of the person phone number. It is also something that is key that will help us to be able to trace the person and I mean a popular eeh.., eeh… landmark of where the person stays. How far the person stays from that point. it's also something that is very key to help us or... In our dispensation or our certain, a popular person or popular name in that locality, can also help us to locate the person in case we are tracing or looking for the person. Eeeh, all these things aside, now our eeh... How do you call it? GPS address, if the person has one at his home, it will be something that will help us a lot to be able to trace directly when these addresses, because I know that is more eeh eeh on point than the other ones. So, if we have the person's eeh eeh GPS address it will do us a lot of good in tracing or searching for the person. And I know that we are not coming back on anything eeh eeh that we are doing in the previous time in terms of technology, we are improving on that. So, ones the GPS address system has come it's come in handy for us to use. So, I believe that these particular things will be able to help us get these people traced in terms we are not getting them on appointment times. So basically, I believe these things will be able to suffice for our tracing.

**Speaker 1** [00:12:18] All right. Now, there are also instances whereby clients give us information, sometimes accurate information, for where they are living. In fact, numbers, sometimes they may lose their phones or the SIM cards along the way. And sometimes, too, before you even get to the location where they give you the not even they even move from one place to the other. So how do we trace or ascertain like how do we trace in order to be able to get these clients back to care?

**Speaker 2** [00:12:55] Okay. So, on that particular question, I believe it will start from how the person eeh understood the condition that he or she is having and how committed and ready the person is in helping to manage the condition. If the person understands the condition, how the nature of the management is going to be. Then the person will be committed. And if there is any loss of contact, I mean mobile contact, if the person is relocating from wherever the person stays, that spirit of commitment alone will make the person come back to give you eeh the new address and phone number that he has or she has acquired to still help I mean caregivers to continue giving care to...

**Speaker 1** [00:14:10] The person.

**Speaker 2** [00:14:11] To that person. And also, if we can get a certain number, that is I mean, either of the caregiver that the client has maybe written in a certain pocket-sized notebook. The prison can easily refer and call back and say that, Oh, I used to stay here, but now I'm no more there, or because of one or two issue. My phone got missing. I have lost this number and then this is my new contact that can reach me or any time that would need me. I believe that would be able to help us to solve that particular problem or that challenge.

**Speaker 1** [00:14:54] All right. So like eeh in case we have do we do the tracing then we get them ehm, how do we encourage them to come back like we've done the tracing we've seen their clients, how do we encourage them to return to care?

**Speaker 2** [00:15:14] Okay. So earlier I've said that if the person understands the condition from the point of information being given in the beginning of even new conducting the test on the person, that is the pre-test information and post-test counselling and all that before the person is initiated into treatment. If the person really understands everything and is committed. Eeh..We will not be having problem of loss to follow up to care. But we are human. we are subjected to changes depending on what we hear going forward and other things. So certain things may sway our mind, our attention and earlier resolve to give the best, I mean, to the care that is being given to the client. Let's, let's, let's, let's assume that by any way, the client defaulted or has been lost to follow up for some good number of time or period as it is in solving every problem. You start from noon to unknown. So, we must get to know from the client what caused him or her to take that decision of staying away from treatment or not coming in on appointment times to get refill and other conversation that will help us know. Even the current state in terms of health how the person is doing. If the person is sincere to tell us the reason, then we'll able to give appropriate counseling to the patient and encourage the patient if they are any eeh eeh eeh, a mistress that to you might demystify or there are some other myths that must be able to correct in the person's mind. We would be able to do that. So, all depends on the reason that is influencing the client to default or not coming back on appointment for the medication. If he's sincere with that, want to help us into counseling and some existing example of people or instances that we have solved in the past that is related to the person's problem currently. Yes.

**Speaker 1** [00:17:41] Okay. Thank you very much. I think eeh I will be asking you the last few questions, and we be will be wrapping up very soon. So, like hmm. So, let's look at something. Do you have an idea? This is just eeeh we want your opinion on this and do you have an idea as to the characteristics of individuals who are likely to default or go missing eeh during treatment?

**Speaker 2** [00:18:07] Okay. Eeh… some observations made are people that from the start of. I mean, treatment initiation. They seem so spiritual. Or, eeh. Have yeah, one one. One is those who seems spritual. At first, they will still be battling in their mind as to whether the condition that they are having it is something that that that has to do with eeh eeh eeh I mean, the body in physical and borders on his or her health or it is something that is spiritual that somebody is trying to eeh eeh inflict on him or her in the spiritual relms. Those people, you may try hard or you try hard to bring them from the state of their mind to the fact that the condition the condition exist (Noise from outside). So, after trying all these things, these people will still be looking forward to a certain eeeh eeh, a intervention that would take the disease at of their body. They wouldn't want this disease to be existing in their body. So, they would want that thing to be taken out or (Interviewer interupted) because they think they are very firm and strong in spiritual matters. They want to put the medication down and challenge their faith that by prayers and by those eeh eeh spiritual rituals that will be told them to do when they do, they will be able get the disease out of their system. These are some of the people that will that are likely to default on their medications or on the appointment diet. Another eeh people are those that still think that the condition that you are talking about, they don't think it is in existent. Maybe you are just trying to tell them something or maybe there is some eeh program that you are doing maybe money has come from somewhere and you people want to get some of the money. So, you want eeh certain group of people to say that okay these people are having so that you can use their number or their data to go to go benefit from that particular program. So sometimes they will also take the medication alright they go and then they will be defaulting eeh on the medication. So, these are some the characteristics of people that for all things being equal, they are likely to default on their appointment times. Ahaa.

**Speaker 1** [00:21:20] Hmm. Okay. alright. So, eehm I mean, let me clarify that. Once you are there prescriber at the facility level, do you think eeh the clinic. Do you have clinic hours for HIV care? Specific clinic hours.

**Speaker 2** [00:21:35] Okay. From experience here, we don't have a specific time for HIV care.

**Speaker 1** [00:21:42] Okay.

**Speaker 2** [00:21:43] Ahaa. Every day, except on weekends, even on weekends, when the situation is such that people responsible for dispensing eeh prescribing are to be called, they are just, a phone eehm call away, we reach out to them and they come to give whatever they supposed to do to the client at that particular, particular time. So, we do not have a setting time that we conduct our ART clinic. It is open from 8:00 in the morning up to 5 p.m. in the evening.

**Speaker 1** [00:22:24] Okay.

**Speaker 2** [00:22:25] Every day.

**Speaker 1** [00:22:27] And when people come like their waiting times do they work for too long time or.

**Speaker 2** [00:22:34] No. We do not have that load. And because we do not have a clinic day where everybody will be coming, we don't have that problem of keeping clients waiting for a long time and other stuff. No, that problem is not in existence here.

**Speaker 1** [00:22:55] All right. So basically, Alfonzo said all the questions I want to ask you, but if there is any suggestion, any omission you want to make with regards to that interview that we've conducted so far. Okay. You are free to do so.

**Speaker 2** [00:23:10] All right. Okay. So, I think basically everything that has been asked is in order and you need to go a long way to contribute to the research that you are looking at. Eeeh to add to one of the points, some people too feel that you are too young, ahaa, to have such a condition and at the time that eeh, you screen and find them to be positive or reactive to the test, they look at themselves and then they see that I am energetic, I look healthy, nothing shows that I have anything of that sort. So, I don't think I have the condition. They may want to go try certain things, some sort of interventions. That I want to roaming to do some investigation at other testing point to see or to confirm or otherwise of what you have told them on their test that you have done. Ahaa, aside that we have to add that people sometimes, are looking for a means or ways that can take the disease out of their system, like how somebody has malaria. He takes a course of ACT and then the person feels fine. So, they're looking for such, so they will ask you, so for how long am I going to take this one? That I will be free of this one? Is it going to be out of my system entirely? So, such people, we are looking for an avenue that will cure them... cure them wholly. So, when people begin with such at the initiation of treatment. There is some probability that along the line, they may default because they are looking for something that will say no fee entirely from the condition as well. And that anxiety that they have is also linked to sometimes the kind of belief that they hold. Aside that, we have also had the challenge of setting appropriate tools, especially for the children. Somebody will be living here, they come, they expected to be given a certain dose of the eeh ARV. But you may not be having that dose or may have run out at that time. And then you would schedule the person to come at another time. The present comes and person doesn't have it. Maybe you have also call your nearest centres to see if they can help you with some of that particular eeh eeh prescription. So that maybe later you can even replace and you don't get it. You may take the next step of referring the client and the that clients and the client not getting that particular dose. Upon those visits and then those time we send in quote and they may, they may they may decide to relax at home. And that is one of the things that I've notice in one or two issues that come, they are children they not getting the appropriate dose. Some of this people to before you know their parents are not alive, they are orphan clients. They are living with a certain eehm grandmother who is also finding it tough to take care of that particular child and the person checking some distances to other ART centres not getting that particular dose for the grandchild. Eeeh will also tend to relax. Gradually, you see the child detorilating. So that is another factor that eeh, make people get lost to follow up. Aha. And that is also linked to eratic supply, or short supply of those medications. I mean, those prescribed of medications. So, in that are some of the little observations that I have made in terms of the children.

**Speaker 1** [00:27:30] Okay. Alright. Thank you very much. And thank you very much.

**Participant 2**

**Interviewer: [00:00:00]** Okay, so thank you very much for your time. We are going to start with our interaction, this afternoon. So when I ask a question, it starts from you. Then it goes and then comes. That is how we are going to move. Alright. So please, what are some of the reasons why individual stop coming back to the AR… to the clinic for ART?

**Interviewee: [00:00:40]** Okay. Mostly, most clients, they believe in that religious believe they will come. They will sometimes too when you call them, they'll say, I went to my pastors church and they say this and that and that and that. So for that matter, or for that reason, [00:01:00] they can't take the ARVs anymore. So religious belief is also affecting the defaulting rate. (Interviewer: Okay).

**Interviewee:** Okay. Stigma, Yeah. Once the person is having the disease, they feel like, um, they feel shy to come for refill after which, We are done with the counseling meeting for the first time. You can leave and then you call the client to come for refill the patient will not come. Um.. because uhm… they fill shy. Because most client even take their let’s say hypertensive drugs at the OPD. So why are they taking only their drugs at one corner? Yeah, so, stigma also affecting the eeeh… the… defaulting rate. And poverty, poverty. Yeah… this area majority of them their work is eeh farming. And then you go to Siana and other places when you call them to come for refill it becomes a challenge. Yeah, they don’t get money to come. So…we have developed a plan. Yeah, so we have interacted with the CHNs. [00:02:24] So when they go for their home visits and then those clients that we are able to reach them, we just inform the client that we give the medication to one of our nurses to supply them. So we have started. Though there are challenges but we still on course.

**Interviewer:** Let me ask, what, what are some of the challenges when you leave the drugs to the CHN to send it to them?

**Interviewee: [00:02:54]** Okay. One is, um, lack of commitment. Some, some are not willing to take those medications along with them while they're going for their home visit. That is the nurses, okay? And then the client too will accept today. But tomorrow morning you call a client that, uh, um, this nurse is bringing the drugs. He tell you that let him stop. [00:03:20] I'll come myself. But meanwhile the client too will not come. Yeah.

**Interviewer:** So does it mean that before the… community health nurse, pick the drug have you engage the client?

**Interviewee:** Yes.

**Interviewer:** Because at times they need, what we say, consent.

**Interviewee:** Yeah.

**Interviewer:** They need consent.

**Interviewee:** Yes.

**Interviewer:** They need to be told who is coming. Just as you said, the word stigma then all of a sadden a different, a nurse is coming, tomorrow it will be a different nurse. Next day to be, another community health nurse depend upon the area they change. This week I [00:04:00] went here, so the following week I’m not going, you should go. So is it that you already made the two to meet, assure them of confidentiality and they are also now withdrawing, saying they don't want them to come?

**Interviewee:** Yeah. starting, we, we engage the client, the client is tested, if the client is tested positive today. So during the post test counseling, we include all these, [00:04:24] that when you take the medication for maybe more than six months, then you are done with the viral load. Then we can supply you with drugs while you because you are at home. But we… we first contact the client before we even tell the CHN when they are going for their home visits. But sometimes they will accept and then the following day, they will reject the supply or medications.

[00:04:58] I should say, [00:05:00] I don't know whether it's the lack of understanding or what, because we seek their consent before we send the, we give the medications to the CHNs to send them to them. So I think it's lack of confi…eh eh, trust, or what. That's why they sometimes refuse to take the drug and they will say they'll come to the clinic by themselves and...

[00:05:26] And in addition to the challenges, um, some patients who also to want to avoid the side effects of the ARVs. Yeah. Well, before we give you the drugs, we first tell you what, uh, the side effects would be. Okay. Yeah. The duration, maybe diarrhea it will not take you more than 4 days. But these people cause um, though we are human. Yeah., you tell the person it won’t take long, so take it.

[00:05:56] And as they take the medication, they’ll be used to. [00:06:00] But once, they take the medication home, they will call him that they're having diarrhea. I want to stop, but we keep on convincing them. But such clients, maybe the next visit day will be um, somewhere around 6^th^ March, but 6^th^ March you will call the client [00:06:19] and, they also tell you I still have the drugs. Yeah, it has not gotten finished yet. Meanwhile, that means that the client has not been taking the medication as prescribed. Yeah. To avoid those side effects, but we keep on encouraging them to take their drugs.

**Interviewer:** Okay. So in situation where you have such patients missing, that is they don’t more come for the art. What challenges do you face as a health staff in tracing those patients?

**Interviewee:** Some are, um, wrong traceable addresses. Some clients too, um, [00:07:00] um, from the initial stage that we are doing the testing for them. They will provide wrong traceable address and telephone numbers. Yeah. To healthcare provider and once they do that, the person will get home and we try to call the number, it will be unreachable. And some, some others too are not in existent, but, um, Yeah. But we have develop a plan before we even do the tests at the initial stage, we take all this information from the client and then um, call the number to see, to verify that this is this client's number. For the traceable address, the person can easily give you a wrong traceable address. [Interviewer: Okay]. But the telephone number, we, we try as much as possible to call to verify. Yeah. Before we even do the test. [interviewer: Okay]. So that, um, maybe at the OPD the client was screened, and then the client was reacting at the OPD, that is one of our entry points then the client would be escorted to the lab for the true confirmatory test. So, once the client get to the ART, we will take the data that that was collected at the OPD and then you also ask the client to confirm the data that was previously collected. [Interviewer: 00:08:34; Okay]. Yea. To see that…(voice faded).

Relocation too. (interviewer reacted; Relocation!) yeah, of the clients. When the person comes, maybe you say, Uh, I stay at Noberkaw, this place. (Interviewer; Okay), you go there then the next day you go there too, they will say he has relocated from that place that he was staying to another place, and that one, too is affecting the defaulting rate.

**Interviewer:** [00:09:00] Okay. So all these points mentioned are patient related.

**Interviewee:** [00:09:05] Yes.

**Interviewer:** But when we come to facility-based issues, is there any challenge that you face with regard to treat eeh, tracing of patients?

**Interviewee:** Yes. One is, um, documentation problem. (Interviewer: Okay). One, once at times we see a client of which the data that you collect from the client may not be accurate. Yeah. At first is we only take the name then the date the client was tested, without any traceable address and other things. So once, the client does not come for the revisit, it ends there because you cannot track the client anymore. Yeah, but that was then. But this time around. I think we are…, we are trying to solve the problem.

**Interviewer:** Do you have any additional [00:10:00] point you want to add to what he said?

**Interviewee:** That's what he said

**Interviewer:** Okay. But I wish to still prob further. Um, if you want to, if you have identified patient who is lost to follow up, [00:10:14] and you want to make contact tracing, do you have the available means? Do you have what is required of you to make contact tracing or conduct contact tracing?

**Interviewee:** Yeah, we have the tools, but how to get that destination be like um motorbike or other stuff, we don't have. Yeah, we don’t have motorbike to track our client.

**Interviewer:** So how do you, how do you do the contact tracing?

**Interviewee:** so we do it um.., we do home visits and then sometimes too we contact them. [00:10:50] Mostly we contact them on mobile phones. Yeah. That’s what we’ve been doing for now. Since we don’t have motorbike to go to other far places [00:11:00]…

**Interviewer:** So you did mention about the inaccurate data collection. Does it mean apart from that, don’t you have any other, we want to find out because, maybe in the course of collecting the biodata, it wasn't taken accurately. [00:11:30] So getting in touch with them becomes a problem. At the facility level what measures do you put place?

**Interviewee:** Okay. In addition to um... Mostly we have duplicates, uh, client information on the e tracker as well as our registers. Um, for instance, a client will be registered, then will be given a um, a unique number maybe A0002. [00:12:00] We have three zeros before the one, now I will also come and bring four zeros before the one, but that client having one name but two different unique numbers. So the client may visit you and then you do the follow up on the e tracker, but still you assume defaulting. Because the same client who visited, um, my, my colleague nurse and then he was taken care of. [00:12:33] So, uh, the client may be lost to follow out in some way, but, um, who will still be visiting the clinic for refill. That was also a challenge. So we are cleaning our data, we started last month.

**Interviewer:** In what way are you clean the data?

**Interviewee:** Yeah, so we are using the traceable addresses and the contact numbers. [00:13:00] So when I say when the client visit the clinic, we update the e tracker. So our folders, that is with 2015, which we cannot, um, contact them or trace them, with such clients. [00:13:15] Uh, we have stopped or just delete them from the e tracker because they didn’t they don’t come for revisit. And then other things. They don't come at all. So once, the client come for revisit, then we updated the e tracker as well as our registers.

**Interviewer:** Okay. Is there any additional, Okay, so what about patients who are traced? Does that matter in tracing patient. [00:13:43] If the patient wait too long, that is the patient is lost to follow up for too long to find out. What made it harder for them to, for you as a health worker, to bring them up in such situation **Interviewee:** [00:14:00] the client long?

**Interviewer:** Who are lost to follow up for long? What make it difficult for you to bring such people back to care?

**Interviewee:** Okay. For such clients, once we have their traceable addresses and their contact numbers, we do so. [00:14:17] And if the client is not, um, a way to come back, we mostly visit their homes and then speak to them. Some, some will have a challenge. because, um, when I took over the ART, um, one guy [client] told me some nurses were demanding some money from them, before they will be dispensed ARV. Yeah. So they, they advised themselves not to visit the unit again.

**Interviewer:** [00:14:46] Okay.

**Interviewee:** Yeah. So with this, we keep on interacting with them. Then we told them everything is free from testing to medication everything is free. And then, uh, we have been able to bring more client back to treatments. [interviewer; okay]. Yes. But at [00:15:00] first, for a whole month, um, we'll be expecting about 30 clients to come for, let's say in June. But you see only six clients who will visit the clinic. [00:15:11] So you are there, you may have the numbers, but they're not coming.

**Interviewer:** do you have something to add?

**Interviewee:** As he was saying, [00:15:23] they, they, when, they told us that, some nurses are collecting demanding money from them. We have been able to advise them that it’s not true.

**Interviewer:** So I want to come in with a follow up question, when they say some nurses demand money from them, that is patients who come for the various ARV, Is it that when they come to the facility for their drugs or when the nurses as you said are [00:15:52] Uh, given the drugs to go and give to them at home that they demand for the money?

**Interviewee:** Okay. That case, I think, um, it was a woman [00:16:00] and she delivered at the maternity ward. [interviewer: Okay]. And then, uh, her husband came around to see her in the, um, she was HIV postive? [interviewer: Okay]. So she was, she was discharged and then the nurse told the husband that um, his wife is having this disease, so uh, they should bring her one crate of egg, eggs. [00:16:27] Yeah. And then that led to that um, lost to follow up because she decided not to come again because she can’t be coming here and then paying money or people will be taking some gifts from them. Yeah.

**Interviewer:** So it was actually the, the wife who revealed that the nurse demanded money from or the husband rather?

**Interviewee:** The husband too became positive. [interviewer: Okay]. I think in 2016. And then, uh, [00:17:00] they came for ART and then defaulted. [interviewer: Okay]. So we went there and then he personally told us, [interviewer: Okay], what happened on that led to the reason why he is not coming for the ARV anymore. [interviewer: Okay]. Then we explain issues to, some things to the client. Then as I speak, um, he had been coming for refill.

**interviewer:** Okay.[00:17:31] Do you have…, do you have something to add?

**Interviewee:** Noo

**Interviewer:** [00:17:42] So should, so should certain individual who go missing be traced first?

**interviewee:** Yes.

**interviewer:** Is the question clear?

**interviewee:** No, please.

**interviewer:** What we are asking is that [00:18:00] we are talking about those who are lost to follow up, and we are saying that, do you think that, uh, once patients are missing? [00:18:14] What about the patient that we are being saying that they are missing? Does it matter?

**Interviewee:** Yes, because when you don't treat those people with the disease, [00:18:27] the client will still be infecting some people with the virus because when you put the client on treatment, it will be difficult for him to infect other people. Yeah. So the client will be viral suppress [interviewer: Okay], while on medication and then it won’t be so much easy to infect other people. [interviewer: Okay].

**Interviewer:** So what challenges do you as a health staff face when you try to encourage patients to come back to care? [00:18:57] Especially the recent patients.

**Interviewee:** Yeah. [00:19:00] Let say um

**Interviewer:** Pause there and let him come in. Yes. Let, let him come in. The what, what are some of the challenges you face when you try to encourage patients to come back to care? That is those who are lost to follow up.

**Interviewee:** Okay. They, they become lost to follow up on the e tracker too. [00:19:24] Because when they do not come for the ARVs, we can't even do follow up for them on the e tracker. And that one becomes a problem for us. Because supervisors, they mostly come around to check on those things too. So when they don't come, it really affect us a lot.

**Interviewer:** If who don't come?

Interviewee: If the clients did not come to take the ARVs, it really affect us on the e tracker too

**Interviewer:** is that the (voice not clear), they not coming and it’s affecting you? [00:19:55] because monitors will come and you wouldn’t [00:20:00] be able to defend yourself, is that the only thing? What are some of the challenges?

**Interviewee:** That's that. Yeah. And one major challenge too is, um, religious, religious, uh, leaders to are inciting the populace. Yeah. Because I think today we decided to call some client who are defaulters.

**Interviewer:** [00:20:27] Okay.

**Interviewee:** And then lost to follow up. So we did that and one man told me that, um, he went to uh, one pastor and has been given “Yezu Mogya” (Jesus blood). So once he thinks he use “Yezu Mogya”, all the viruses is gone (Laughter from both interviewer and interviewee). So he will no longer come for revisit or come for the ARVs anymore. [interviewer: Okay]. But we keep on speaking to him. Yeah. As time goes on.

**Interviewer:** So let me come in, so now because of the “Yezu Mogya”, how does it [00:21:00] affect you?

[00:21:01] It's really affecting my defaulting rate. It’s making it higher. but I can’t also delete the person from the e tracker. Yeah, it is taking my defaulting rate, always going up. because they don't come.

**Interviewer:** And that is difficult for you?

**Interviewee:** Yeah.

Interviewer: So, um, [00:21:25] for those who do come back, do they give any reason why they come back in case the person is lost to follow up? You have contacted him or her and the person come to treatment. (Interviewee: Yeah). And then give any reason why they decide to come back?

**Interviewee:** Yes. Once, the client comes, we ask the main reason that was preventing him from coming for the ARVs then we try to deal with that issue. [00:21:55] Yeah. If it is, um, it's about [00:22:00] money or transportation, we see how we can help the client.

**Interviewer:** In, in reality, does, do you encounter some of those that the patient will give you reason, like because of this, that he was he or she was those before that?

**Interviewee:** Yes. (interviewer: Okay). I think last two months, one client is um… in a town, in the Bibiani road. [00:22:25] (Interviewer: Okay). So, She told us she can’t always be coming here for the ARVs, so we gave her six months. (interviewer: Okay), and then we inform our CSOs. Um they came here and told us that, that if the client needs help in terms of transporting the ARVs to the client, we should inform them. So we, we call them and then we inform them.

**Interviewer:** you said you inform your who? (CSOs) [00:22:53] Okay.

**Interviewee:** So they took the client number, but we sort permission from the client first, um, in that this person will call you [00:23:00] and try to interact with you (interviewer: okay), and see your challenges and then try and help you out. So, they, they told me, they gave me feedback that, um, they will give, they'll pay the transportation.

**Interviewer:** Who might the transportation?

Interviewee: The CSOs. Yeah, they, they will pay for the transportation for now once they're working with us. (Interviewer: Okay). For some time. Maybe if the client is due for viral load, they will pay the grant to come take the sample. Then if they also due for revisit, they will pay for the client to come. Because they also bringing them back to treatment. (Interviewer: Okay). Yes.

**Interviewer:** But on that, is this something sustainable?

**Interviewee:** No. That's why I told you that they doing for now, because it's a contract. (Interviewer: Okay). [00:23:51] as time goes on, (Interviewer interrupted: they voluntarily say they will be sponsoring? Yes.

**Interviewer:** So looking into the future, if they are able to continue for some time and you [00:24:00] realize that you are having patients coming back to treatment and you think it is something sustaina.. um that should be maintaied. How do we sustain it? They may not be able to continuously sponsor their transportation always, [00:24:18] and those things. Is there any way you are? (Interviewee: Yes). You thinking seeh it'll be of help?

**Interviewee:** Yeah. This time around, we make it clear, clear to them that, um, taking the arvs will suppress the viruses it will help, uh, the client then to help me as well as well as the society. So we tell them the importance for them taking the arvs. Yeah. So that if as time goes on, the the contract has been been terminated, [00:24:57] it wouldn’t be something like we have disappointed or what

**Interviewer:** [00:25:00] well, do you have any additional other.

**Interviewer:** [00:25:11] You as a health worker, do you have any competing responsibilities, because I don't know whether you are specifically assigned just to the art, HIV clinic you're responsible for, that one alone. Is there any other responsibility that, um, interfere with your work as, uh, an nurse?

**Interviewee:** At our ART, we have two units over there. [00:25:47] That's the mental health unit and the ART units and the nurses that place we are plenty, we more than eight or so at, at our unit [00:26:00] units, we are there with mental health units. (Interviewer: Okay), ahaa, so when the persons come, sometimes feel shy because of.

**Interviewer:** So, so how do we, how do we resolve this issue so that you will have a, an isolated place? [00:26:17] Is it possible?

**Interviewee:** I think, um, we spoke to medical sup and then he met with management and then they have decided to relocate the mental health unit or the art unit. So that for privacy sake. Yeah. So for now that’s what…

**Interviewer:** But it's not yet. It's something that is yet to be done.

**Interviewee:** it’s yet to be done. (Interviewer: Okay).

**Interviewer:** [00:26:50] So do you give. Or do you, Do you always receive any incentives in terms of your contact tracing? [00:27:00] When you make contact tracing, do you receive any incentive with regard to where you operate? That is the, from the facility management.

**Interviewee:** Yeah. You are being given some amount to purchase data and then do follow up visits for our clients, and they also give us money for air time. (Interviewer: Okay). And then our partners that’s JSI, they also support us with the data and then airtime. [00:27:33] Yeah. The rest diee.. and then we also go for workshop.

**Interviewer:** how often does this incentive come?

**Interviewee:** For the facility one, um… it's every three months first quarter, second quarter, third quarter and fourth quarter they’ve been given us. And then for our partners, too JSI, they do it every three months. Yeah, so they do it as… the same. (Interviewer: Okay). Yeah. But sometime too, they combined, uh, all six months and they supply to us.

**Interviewer:** [00:28:00] Okay. In the beginning of the year, second quarter, or? **Interviewee:** It’s from first January to June. (Interviewer: Okay). Then they will supply us with data, and then July to December too they will supply us. (interviewer: okay, okay). So they do it in advance (interviewer: okay, alright). **Interviewer:** [00:28:24] But when we started, you were talking about little time, and then I mean, means of transport. Um… when you identify a defaulted patient, how do you move to, in case a person is reachable. How do you move to give the person that, I mean the person contact is properly given, the person address is properly given and you know where he or she stays. [00:28:54] How do you move there?

**Interviewee:** Okay. We normally go there with the [00:29:00] mental health people, when they're going for their home visits. Yeah. They are having motorbike. (interviewer: Okay). So we pedge them, and then we go. So sometimes too, (interviewer: Okay) Um, We give those, the client information to the CSOs to also help us to bring those clients back to care.

**Interviewer:** Okay. So let me make it, So in that case, where… is it possible that maybe the mental health people will not be going to the field at the time that you have [00:29:38] made your target to go and meet the client and maybe try to convince him or her to come to treatment. Do you always try? Is there a possibility that their bike can be given to you? And if it is given to you, who does the fueling for you to go?

**Interviewee:** Okay. [00:30:00] When you need the motorbike to go for our home visit, put it in writing. (interviewer: Okay). Then we submit it to administrator for him to approve, then Med sup will also endorse, and then we raise a memo for fuel (Interviewer: alright). Yeah

**Interviewer:** [00:30:18] So when do you start the tracing?

**Interviewee:** Yeah. Once we detect the clients, has defaulted or has been lost to follow up, even be it two weeks or defaulted for more than three months. Once we see them, we start tracing the client

**Interviewer:** [00:30:39] That’s fine.

**Interviewee:** We try to pull out the folders, and check the due dates, whether the person has defaulted or not, and we put the person name on paper and after which we start calling them.

**Interviewer:** So you say, so based on that, what time do you also set off?

**Interviewee:** [00:31:03] The time we start (interviewer: after you’ve identified a defaulted person). Yeah. We do it weekly.

**Interviewer:** Is it in the morning, afternoon, in the night, in the dusk?

**Interviewee:** Yeah, in the morning. Morning. Every Friday morning, we try to meet the e tracker to generate those who are due, and then those we would be expecting next week. (interviewer: Okay). Then we pull out all those folders, then try calling them. Some may come and then some will also tell you I will be around next week. [00:31:34] So those folders will be separated from those who have already come for their revisit. (interviewer: that’s good).

**Interviewer:** [00:31:45] So in case the person come to you, How do you get, what are the type of contact information you need from him or her?

**Interviewee:** Okay. [00:32:00] we take their mobile numbers, then we take their traceable address, at time too, the land, the landmark. (interviewer: Okay). Being, yeah, maybe their house is closer to a public school or, or a toilet facility. We take all these details (Interviewer: Okay). Yeah. These are the two main that take from the clients.

**Interviewer:** [00:32:32] Please, number one. Do you have any other additional…?

**Interviewee:** We, we take their phone numbers as my brother said, the exact place they live to contact them. And in addition to, we take their relatives contact. If the client…, like it can be it be the client husband in case of, um, in case we are trying to reach maybe the wife and we are not [00:33:00] getting him, we try to contact the, the husband. (Interviewer: Okay). Yeah.

**Interviewer:** [00:33:07] So you, we've already talked about how to, how to encourage the patient to return to care. [00:33:17] Isn’t it? (interviewee: Yeah). How do you encourage them to come back to care?

**Interviewee:** Yeah. We made them aware of the importance of them taking the medication and …..(voice unclear) demonstrate to them, um, When you have the virus at the initial stage, the um, it has some colors. (interviewer: okay) Yeah, we have some beads in a bottle. The beads, we have the red colors and then, and the black colors. [00:33:48] So once you have the disease at the initial stage, it represents the red, the red is the virus. And then, um, your health is the black one. [00:34:00] So while you take the medication. They red, vanished, then you have more blacks, meaning you, you are viral suppressed and that means, It's not possible to infect others. So we have all these pictures to demonstrate to them whilst we are doing post test counseling. [00:34:19] And then while, um, they being brought back to care, we demonstrate all these to them, for them to see. And furthermore, we, we tell them the importance of the viral load, because when you take the arvs, the more you take it, it will suppress the virus in your blood.

**Interviewer:** Thank you very much. We are almost at our last end. You want, you made a very important point with regard to you predicting who will go missing, that is lost to follow up or can easily become a defaulter. [00:34:56] Can you emphasize on that? How do we [00:35:00] predict who is likely to get missing that is lost to follow up?

**Interviewee1:** Okay, we can use the e tracker. (interviewer: let number one come in). We can use the e tracker. (interviewer: Okay). To check those who are overdue. (interviewer: Okay). And pull out those folders to check those people who are overdue. Because let’s assume I came today and my next visit, or my, my refill date would be next two months. [00:35:31] and when the date to, we decided to pull out those folders and call them. So when they don't come, we put those folders aside try to call them. Uhuu…

**Interviewer:** [00:35:44] Any other?

**Interviewee:** Yeah, I think, um, their next revisit date we make all available registers as well and then the folders. And then put it also at the comment section. (Interviewer: Okay). [00:36:00] So once you open the folder and maybe you're going through. When you check the last visit date, you will see it at the comment section. (Interviewer: Okay). That, um, the client suppose, the next visit or next refill date at the comment section. [00:36:16] if you put there. Then we add when the client will be due for viral load as well, all at the comment section. Then we have it too in our dispense logbook. Then, uh, our ART register as well. The e tracker will show how to get it. And we also have current status at the back of the folder. That’s if the person is due for viral load or lost to follow up.

**Interviewer:** So we, uh, determined the… briefly, we have interacted and we've gotten to this stage. [00:36:56] Is there any other issues, [00:37:00] um, view that you think you have not brought in or we have not asked in this deliberation? If you want, if you want to further bring or add.

**Interviewee:** [00:37:14] I think that's all, uh, because we are, we have bring you whatever that is going on at the clinic. Yeah. I think that's all the challenges.

**Interviewer:** [00:37:34] You see, uh, I still want more clarification. Let’s say if you have this client coming, you’ve put them on treatment. Let’s say ten, how do you predict that this one will get missing before that?

**Interviewee:** [00:37:55] Okay. If I understand you well, um, Lemme use your figure 10 clients on treatment, um, then maybe we have scheduled them for maybe we next month 15^th^. Um, mostly we have some clients who are already defaulted. So we know those clients already, and then we normally put them at the comment section. So those clients, we don't give them, um, drugs which will take them for long.

[00:38:25] We’ve trying to test them maybe at first we give them six months. Then they defaulted one year, then we have tried to bring them back to care. (Interviewer: Okay). So we will give you one month. (Interviewer: Okay). So as and when you come. (Interviewer: Okay). And then we increase the number of months for you. (Interviewer: Okay). Yes.

**Interviewer:** So meaning you are predicting that this person, if I don't monitor this person, he will be lost to care.

**Interviewee:** Yes. It has been some kind of punishment that we give to the client.

**Participant3**

[00:00:29] So as a health worker, what are some of the challenges that you face? When you try to encourage patients to come back to care,

[00:00:42] okay. Basically, I think first of all, believe is that they have some, should I say as I put, okay. As I. The person will come, start with the person, open the person going through all these counseling. [00:01:00] I said start with the case environment. They get back. Mm. They have different thought. They what people see.

[00:01:08] Okay. And the negative impact of, should I say hiv? Mm-hmm. , Most of them think is. Something spiritual. Okay. So although the person have started treatment, but I get back into the community and they, they look good. Okay? So I was already confident to come back after some come. So in that case, how do we do away or try to, um, ensure that they cannot believe that people have, with regard to.

[00:01:40] Is discouraged among, especially the clients. Okay. Luckily, I think we should deal, It should take education. Okay. On having You don't have any issues. Okay. [00:02:00] That's a key part of it. Okay. They think HIV is a disease, okay? You them understand and having hiv, you don't have any disease function. You're not sick, okay?

[00:02:12] But you find the virus you okay? But if you don't go home with a medication that, okay? If you're able to pitch between these two, I think it'll help a lot. Okay. Secondly, I think we should deal with, uh, should I say religious leaders? Okay. I think they're part of the whole situation. Okay. Although us really go to talk to them.

[00:02:34] Understand. But any time, you know, you're in Africa mm-hmm. and believe this, I think part of this, So should I say this Hmm. To themself will be explaining to them. Mm-hmm. . So they is understanding data, but I think they understand more than being house with them. So who have mine in mind? Maybe we felt okay cause we are paid to do this work.

[00:02:57] We come out to see meeting we want. Okay. [00:03:00] But if the people did believe the pastor cut and say hiv and I think that one help them to come. Okay. And those who continue. Okay. So you, you, you started by saying that the leaders are part of the program. Is it that they do contribute religiously? Does the religious leaders do them, contribute to.

[00:03:23] Given maybe some negative information about the disease? Yes, Yes. Okay. But they always tell them that if I have good look along with it. Yeah.

[00:03:39] Okay. That means someone put the sicknesses or maybe the infection for them. Okay. As long as they change. Okay. Even today we have that experience. explain. The man is from, uh, it, Natalie, some reason. Okay. [00:04:00] He's not from this side. Okay. So he came for mm-hmm. . So he just came and attending to her and to him rather.

[00:04:07] Mm-hmm. So they started, and when you come try to engage, you smoke, you know, maybe in decimating. And this one was saying, let me read.

[00:04:21] Oh, okay. They have an issue on London. Other person bought this kind of sickness or infection. Okay. For.

[00:04:35] Okay. And he started laughing. So she help. Mm-hmm. , I saying You, But I'm going to four places to activate and they're saying the same will be time. I, Okay. Four places like this, ly. Underst different. Just all saying the same thing been. Okay, so [00:05:00] I'll just try to put in that if these for people. Mm-hmm. , we're having knowledge about HIV and they're able to tell, say, Oh we money.

[00:05:10] Mm-hmm. . Okay. They take it from there because they believe then they believe. Spiritual tennis. Okay. Okay. So

[00:05:28] if I may come in again,

[00:05:33] once a vision is. That is in this case, loss to follow up. Loss To follow up. What are some of the challenges you face in tracing? Okay, before we initiate, we, we take addresses. Okay. And we, we take from numbers. But you know, Yeah. Two false, The stigma. Mm-hmm. [00:06:00] I start to HIV even before you glance, unless he give the consent.

[00:06:05] Okay. At home, Okay. You visit my doctor and difficult, So they always need to give you consent before you, So basically we depend on from the contacts to call them. Okay? That is our delay. Always comfort to get there. Somehow tell, oh, you do verification. We always verify in front of the person. The person is in town.

[00:06:30] The letter is good. You can pay the person. They get back to thes. Okay. They want become another. I see. So pet that basically. Mm-hmm. , I miss we time get to them in their houses. But maybe one will be, maybe even the transportation will be an issue to have. Cause. Is, and they don't give that grace you always want.

[00:06:53] Mm-hmm. , we had a case like, uh, they say Joseph photograph always, [00:07:00] people try to give the biggest town closer to the village, to you and even well, village. Okay, so this person from Jesse Fre. Mm-hmm. . So we interrogated

[00:07:14] Oh, bar I am. Mm-hmm. given a what? What, Okay. Not where this person is coming from. Just, Okay. You even get so in case you want to. Go there. Mm-hmm. and see the person on your own. Mm-hmm. , you get to drop and you never find this person. I see. Is that all the challenges with regard to tracing the contacts of sad people?

[00:07:50] Yeah. As I talk about the phone calls too. Mm-hmm. , uh, phone calls, I think that one was, Yes, we are changing to help, but most numbers are not going through [00:08:00] because of the network challenges. One where they're and these people, Oh, changing phone numbers Okay. To is an issue. Okay. Person. We always try to ask Last.

[00:08:16] I'm now so, So what do you. To resolve that issue of patients who provide their numbers to you when they are on their drugs or, uh, in case they want to change. Is there any possible means that you can do so that they can reach you? Or encourage them, Always read back to you that I'm going to change my number cause of the relevance of taking the drugs.

[00:08:45] Is there any way that you can encourage them to either always maintain on the number that they have given to you, or if they're going to change their number? They can't get a tax. Yes. I think [00:09:00] basically, and this thing came up, I think last three years or so. Okay. The supply of drugs was irregular, so we are doing this always when they come, we write our contacts and the fact of the appointment test.

[00:09:15] Okay. With, they aim that before they sitting from the, we don't them to come, not get a drugs. Okay. The cost, we are just trying to go away with the costs. So we always write that our numbers behind the are. Mm-hmm. . But before you come, Okay. Call less before. Okay. So most people were calling mm-hmm. . So that, that was making us to know that, okay, this person has sent the number mm-hmm.

[00:09:42] or give a different number to call. Okay. So that's what we busy on. Mm-hmm. . So if you call, Okay, we know this number well, my sister number. Mm-hmm. . . Then you put that to a lease. Okay. You, not your own, but you add it to, Okay. Uh,[00:10:00]

[00:10:02] so can you think of any situation or experience, does that make it difficult for you as the health worker to find missing patients? Any situation or experience that make it difficult for you to find missing patient? Um, like some of me, like the person contact are going to, and that address given to, it's not the right address to put it.

[00:10:29] Okay. In situation it's always difficult. Mm-hmm. with that unless the person comes again. Okay. That's what you can update. Okay. Or last? Yeah,

[00:10:46] be outside and, Okay. Then we got the can give you maybe the new number. Okay. And maybe update address. Right. Right. So, So do you see contact raising of patients to be [00:11:00] a matter of unsen? Why? Yes, Because we're not getting them. We want them to be on drugs, always our contact with us. They're not giving the right addresses, so to put ahead we not reasonable for, And some instances, it's not that they are lot of drugs they mm-hmm.

[00:11:23] But the business side, they registered. Okay. And already they come and we update on the east tracker. Mm. If the person goes for drug, maybe from a different facility. Okay. And it's not updated on Tracker? Always. We see such a person as a default. I must follow up. Okay. Which is not so mm-hmm. , so I don't know if you can bring that if the person go for help as well.

[00:11:47] People go for drugs. One example is, uh, a lady came to, So lemme put. She will say, You already come to visit, [00:12:00] but they say with emergency. Mm-hmm. , they go and some are so careful about their health, so they go for the medication. Okay. But people at the other sites give them drugs, but they don't go the E tracker to.

[00:12:15] People on the other side like, Like maybe as , the person is from here. The new place where this is? Yeah, going for the drugs. Okay. Hang. They need to update because the persons feel comfortable. Okay, so on the system you see that the person is not for new. The person has gone for the drugs and taking the At a different facility.

[00:12:34] Facility, but cause it's not updated on the system this year, person lost full up. But in reality, the person taking the drugs, Okay, I have an experie sex. The person when they think drive is says, Goodness, But the person that comes here has lost tell you so, because no is also because the person hasn't come to you over and over.

[00:12:58] So I want to [00:13:00] understand something. Mm-hmm. , they're lost to fully abnormal. Is it a system that automatically captures Yes. As not you? We can eat Manuel, but how we are pulling data from the E tracker, I'll tell Is that because you didn't. Okay. Okay. If the person to you and after to the person you don't, so that's a.

[00:13:24] Okay. And put the food aside, you know, update the system will tell you that the person is not So on that experience you were trying to give that the person went to a different facility. Good. Went for medication, The person wasn't updated, the person was sent, who wasn't updated. Okay. So how, how, when did, how, in your interaction with the person, how.

[00:13:51] Were they able to, you know, capture the class that we are given to the person or they just gave to the person to go, you see? Mm-hmm. With, [00:14:00] um, hiv, the issues that we don't want to cause, we don't want them to, shall I say, default. Okay. For where the person comes with it. Appointment, and you need to give drugs to the person.

[00:14:13] Okay? That was what we did before the tracker came in. So the ER is in now to tell you that this number of people are lost, but if you don't enter on E tracker, you never see it. Okay? It's already tell you they're to follow up. They're not to follow up, but if you enter, they're all lost to follow up. Okay?

[00:14:32] So in this the Al, mm-hmm. , take the drive. Massive people give the present. Okay. But you know, maybe take the plane to go hit tracker to update. Okay. Because we saw it, I saw this early, so we were doing that. How you, I want.

[00:14:58] How do you [00:15:00] get to detect that situation where the person was given the, the drugs for the six month continuing six month, unless the person held back to you that used to identify that this person was at this place for number, number of periods. So when we said we are trying to, we are trying to avoid all these things, so what we do is.

[00:15:26] Right. Their unique identifier number, they unique number. Okay. And the site A number. Okay. So we were trying to write both numbers and top of the, Okay. So that time they comes to you, you can just update. Okay. Okay. So if people were, if were, we agreed and we were doing that, I don't think we see people.

[00:15:54] Maybe we'll get them by, but, Cause we're not trying to repeat [00:16:00] distance. Okay. I think that concern is properly. Um, So let's move on. If you wait too long to find the patient, does it make it harder for you to bring them back like the person you presumably, or in reality, the person goes to Fuller, you've waited over and over and over, and you have not been able to get the person.

[00:16:34] How difficult is it for you to get site people back? Those, maybe those who have given, given our can come to their houses, but most of the time we deal with community health nurses. Okay. As they're going the community, the person give us their address. Okay. So it be, go for their cwc. At least you can sit on this person.

[00:16:56] Okay. If the person is there, okay. But the [00:17:00] is already difficult. Proper address. Okay. Then this one become a challenge. A challenge. Mm-hmm. . Okay. Okay. So in case you find them, in case the person is lost to follow up and you find them, I just want to understand mm-hmm. in your situation when we get there.

[00:17:22] Damn, Uhhuh. Do you, Is there, How soon is it? To find citations, like the person is assume or in relative loss to photograph and you try to make contact. How soon are you able to get such people? Okay. Like then you track yourself. As I said, it tells you people are lost to photo depending on the period you go.

[00:17:45] So with time we try to pull the data. Mm-hmm. . Pull the data to see time. We have a be able to do some, some things that are, it'll prompt you that this person is follow [00:18:00] because depend been 30. Okay. So that prompt we those. Okay. We can get completely Don . Mm-hmm. others to, it may not even go. Okay. Ahead. Okay.

[00:18:15] So those who we can get on. That's what that bring the co message on board. Okay.

[00:18:25] So, okay, so in that case, um, you try making contact. Yeah. Do you have, um, though you've indicated that you always have internet and sorry, network. But the, those calls you have been making, do you have any challenge maybe in the necessary, uh, resources, like the calls to be making that? Or is there any provision that you should, or is your own initiative that you are put in place to do [00:19:00] those things?

[00:19:01] I think it's our whole initiative. Okay. Uh, cause data, you know, In Generalism, not data counselor. Purpose. Okay. And when I give you data general, this is what you're for. So, So there's no specific credit for, For calling? Yes. Okay. Okay. Well, what about it in general, making contact? Tracy, what? Do you have any challenge?

[00:19:25] Like maybe me, So yes. The means bag for, Yeah, the other thing is a telling. It's a telling. That's what, basically we try to be first try to write behind. Okay. Okay. If one extent to bring, you can't get over. So we always try to take the opportunity to ride behind them. Okay. If you're going to this community, please start to up all this place.

[00:19:48] And do you have any challenge in them? Maybe accepting, you know, is additional role that they have to play. They, they're willing to. Okay. They're, it's not that many, right? So people are going to [00:20:00] young community here like this, okay. And they're gonna one patient. So in, in, in tit, do you think that people who are lost to follow up should be traced?

[00:20:12] Yes. Yes. Okay. What about those who are maybe very sick besides that, Do you have any situation of that sort that yeah, we help people who can work. And, uh, when we started, when we started with them, they were move to coming from, they would take them down by other sickness. Oh, that's not necessarily hiv.

[00:20:37] Okay. So, so with that, we actual relative mm-hmm. you trust? Okay. So one I know from Community B like this, always let a child come for it. Okay? Okay, I see. And so the challenge has been the value. This another issue, Uhhuh. That means the we need to go there to, [00:21:00] Okay, so, So these are special cases. Yeah, no, we don't have so such special cases.

[00:21:08] Definitely you have to make the effort to go. So do you have the support of either the district, from the district health that is, or your facility to get in touch with search people because you have to buy all these, go to pick the sample from them. Cause they are special cases or there's no, no special.

[00:21:27] I support that actually most accessible for them. So, Try to be smart in the system by getting those there. Okay. You know, this is obviously at least a month you go there. Alright. So we always try to ride behind, but some that one is serious because maybe most of not alls are allowed to go. Okay. So the community but day, but going to is another issue, right?

[00:21:52] So maybe unless we have time to talk with the lab, maybe we came, we one guy. Okay. [00:22:00] So that's great. What are some of the challenges you face when you try to encourage patients to come back to care? That is you realized that the person is. almost getting to, uh, to default or is lost follow up. Totally. And you have been able to trace, like you were saying, you've gotten to the tracker, you've realized that the person is indicated lost, follow up.

[00:22:25] What are the challenges that you face? To encourage people to come back to care in case they have all actually been lost full up. If I understand that was they were lost full up. Yes. Now we'll be able to contact the them. Yes. And you want to now encourage them to come back care. What are some of the challenges?

[00:22:44] Well, maybe. Let me put it this way. Anyone who is just to follow up apart from how me this case. Okay. Which is maybe the trouble taking somewhere who start tracker. Okay. Certain people should meet them. They everyone [00:23:00] have a reason. Mm-hmm. , such as, as I talk of the, this religious leaders. Okay. Okay. Person. I started already.

[00:23:06] Already. Okay. Okay. Person to take time and talk to them. Okay. Try to let them understand that no, there is no sickness now. Mm-hmm. , so don't think cause you're healthy, dad. Okay. The viruses are in there and I always tell them that these barrels, if you're in there, we can never think about it. Okay. The best can is just suppressing.

[00:23:30] Okay. And if you tell them they are top the. Come to the understanding they, Okay, this is the situation. Okay. I know I need mercy counseling at times, some people counseling is, they don't put it out to them in the reality of this whole thing. Mm-hmm. , but every person, just counseling. Maybe I came in to offer self-test.

[00:23:50] Mm-hmm. for, to test me I, not sick, sorry. Counseling in the decide of who or help anyone who initiate going to cancel them before. [00:24:00] Okay. Okay. So if you tell the person, Oh. Maybe I'm talking with you. Try to counsel you before you start and I'll tell you, Oh, we are here. This your sickness. This your sickness.

[00:24:13] But the person sitting now is not sick. Okay. But we'll be able to identify the virus in the person. Okay. Now this person is not sick. So if you put a pocket of your mind, Oh, get back if they came back home mm-hmm. you, but sick. Okay. And I tell them mm-hmm. , the things are mostly for me to take this medication or come for the medications.

[00:24:35] Mm-hmm. . But if we make them understand that, Maybe you may come with some sequences or some sizes before we can test and know mm-hmm. , but when we get able to identify the viruses in you mm-hmm. , maybe we should go with the sickness itself and tell that you have the virus now. Okay. You are not sick, okay?

[00:24:55] You are not a stage. Okay? So whatever we're doing now, [00:25:00] he said about you not getting to the East Street. Okay. The one, they can't take them out. I always tell them, I know other people have on long child On On long child. The virus is there. Okay. The best way you can, you'll able to know that maybe your suppress is doing viral testing.

[00:25:16] Okay. When you use this antibody testing to do mm-hmm. . It'll show. Okay. Because everybody have be able to identify that. Okay? So we just need to make them understand that this is two different things. Okay? You are here now. We don't want you to get here. Mm-hmm. . That's why we were on this. Okay? So all not mm-hmm.

[00:25:36] but we don't want the virus to make you. Okay. So in situation where you have been able to encourage a person to come. Do they? Do you what? What is the reason why those people will want to come back? The person is lost to follow up. You have identified the person, you've encouraged him or had to come back to care.

[00:25:57] The person has a agreed to come back [00:26:00] to care. Is there any reason that they give as to why they are coming back to care? Yeah. As I said myself, say mm-hmm and some people there say so, so sorry, but I. Point is with me, but I want to understand you. The person has agreed to come back. Why would the person agree to come back based on the, maybe the visits or this place you give about the Okay.

[00:26:25] Commission. Mm-hmm. that you're taking for life. So if you're being counseled, I always make you understand that you are taking it for life. Okay? So most of all, So that's, I started with you. Even when they travel and there for two days or three, you don't have medication to call you. Mm-hmm. . So I displeasure.

[00:26:46] Okay. My drugs are shorter. It's two days now will have any effect on me. Okay. So people who understand the counseling and they're willing and they understand the whole situation. Mm-hmm. , they're willing Okay. When they come, [00:27:00] But we have a, a system put in place that mm-hmm. , you're lost to follow up like this.

[00:27:05] I, you consider this. Okay. So as we, we've talked and we all agree, we will not start by giving you maybe multiple months in this person I, multiple months. Maybe you can give you the. Six months. Cause the person, first of all have just to follow up. Okay. So with that, we'll talk to you and we start maybe giving you one month or two months Okay.

[00:27:29] To be able to know your adherence. Okay. Okay. Before maybe you do the, then can give you maybe more of the drugs. Okay. I had. So,

[00:27:48] um, you, what, what is it? Is it necessary that is there the need for us to initiate? And also always encourage the [00:28:00] contact tracing of the patients who are lost, Fuller. Uh, Yes. Okay. Say we need to let them understand. Mm. You're not picking, maybe one or two things may come up. Okay. And you may not even come.

[00:28:16] Okay. But with that, Okay. And one issue, maybe just to add it just came up and some of I of transportation. Mm-hmm. . The person will come, should I say. Okay. Period. They didn't come the period, they come engage it. Mm-hmm. To know what is wrong. Mm-hmm. Oh, in my asylum. So we are trying to be this way, but yes.

[00:28:45] But you about mm-hmm. Know bro. Okay. But most I see we to do it that way. Okay.[00:29:00]

[00:29:00] Okay. No.

[00:29:05] Okay. But most of them so far, they will be. I see. I see. As a health worker. You, I know you haven't been specifically assigned to, uh, the a RT clinic. You have some other responsibilities. Do those additional responsibility interfere in the effectiveness of operation of your web as an a r t net? Uh, additional responsibilities that interfere him.

[00:29:40] Okay. I would say not really. Okay.

[00:29:46] We don't do this. Okay. You know, so facility like this, that, or each related kids need to come maybe on Daisy. Okay. We are not doing that. You are not doing that. You're not doing that. Okay. Because sometimes we, and if you do that, [00:30:00] they're not even come. Just imagine someone comes. I see. And we are together.

[00:30:03] Uh, I don't mean you not mean . You come. So we don't do that. We don't do that. You come for, we feel we take care of you. You go. We also advice and give you even six months. Don't wait for it to get from us. Okay. Even when you are left the last month, you can come. Okay. Another still here, running here. I see.

[00:30:28] Okay. So in terms of contact tracing, are you always giving any incentives as a contact tracer that is with those full, are you giving any incentive, Incentive, incentive? Listen, . Okay. Okay. All right. Um, that's okay. So how do you predict that a person is going, uh, [00:31:00] as a defaulter that is, is likely to go missing?

[00:31:04] How do you predict that the person is like, To be lost to full. The Okay. Um, you know, we have uh, uh, full of dates. Mm-hmm. , so after person sometimes today, and I take care of person. Okay. Based on the vacation giving. Okay. I need to estimate the next time. Okay. So this date always help to move the person.

[00:31:29] Maybe after a month you're not the person. Okay. Sorry. The person is lost to full up lot to full up Is lost. To full up. Okay. So, so I want to understand the person has come today. I have, I have come today and I, I have been canceled and I have to be on the drugs for some time. What, how do you know that if the person, I am getting closer to a point where I am [00:32:00] likely to be lost?

[00:32:00] Fuller? That's, I definitely did this from. Okay, so, so now the 28 days from the start of the, from the last day home, the medication gets finished. Okay. Okay. 28 days. We don't get you done first of all. Okay. Okay. Alright. Is there, do you have any additional, um, information, experience or point of view regard to patients, loss to Fuller?

[00:32:28] Yeah, uh, um,

[00:32:32] I don't. We, we need to all come to the end. As already mentioned. People, uh, or medication, they are, should I say, goes to fill, which are not to, So the issue I wish we should all come on board so that any time, any client cloud versus at your facility for refill, we need to [00:33:00] try, go onto eat. And update it if we try to do that.

[00:33:06] Most of this laws to follow up will not be the laws to follow up site. We know it may be an artificial loss to follow up, but when a system will tell you that this person hasn't. Taking drug or this number of period based, then the person wants to follow up, but maybe reality the person taking back elsewhere.

[00:33:25] So I think we should all come on board, all sites, every time we do with, we should go on each track and update and best update so that this artificial follow up with Bill, which, Okay. Thank you very much for participating in this particular conversation. I'm most for.[00:34:00]

**Participant 4**

**Interviewer:** [00:00:00] What are some of the challenges that you face when you try to encourage patients to come back to care?

**Interviewee:** [00:00:13] um okay. Basically, I think, first of all believe is that they have some should I say believe, (interviewer: okay), in that the person may come you start with the person upon the person going through all these counseling, I said start medication, but when they get back, (interviewer: Mm). They have different thoughts, per what people say. (interviewer: Okay).

And the negative impact of, should I say hiv [00:00:39] (interviewer: Mm-hmm). Most of them think is something spiritual. (Interviewer: Okay). So although the person have started treatment, but they get back into the community and the they look good. (interviewer: Okay). Eheee, so, it’s always difficult for them to come back after all, I am fine.

**Interviewer:** So in that case, how do we do [00:01:00] away or try to, um, ensure that the kind of belief that people have with regard to HIV is discouraged among…, especially the clients.

**Interviewee:** [00:01:14] Okay. Luckily I think we should deal with… we should intensify education on hiv. We should let them understand having hiv, you don't have a disease yet. (interviewer: Okay). That's the key part of it. (Interviewer: Okay). They think HIV is a disease, (interviewer: Okay). You need to let them understand that having hiv, you don't have any disease, and should I say you're not sick, (interviewer: Okay).

[00:01:40] But you find the virus in you, (interviewer: Okay). But if you don't go home with the medication, that's where the disease may set in (interviewer: Okay). If you're able to breach between these two, I think it'll help a lot. (interviewer: Okay). And secondly, I think we should deal with there…, uh, should I say religious leaders? (interviewer: Okay). I think they're part of the whole [00:02:00] situation. Although I you go to talk to them, they will [00:02:02] understand. But any time, you know, you are in Africa (interviewer: mm-hmm). and believe is, I think part of us, (interviewer: Okay). So should I say if these religious leaders are on board, so that they themselves will be explaining to them. (interviewer: Mm-hmm). So if the religious leaders understand it and talk to them, I think they will understand more than we the health workers. Some people have mine in mine that [00:02:21] maybe we health workers because we are paid to do this work. We come out to say anything we want. (interviewer: Okay). But if the people they believe… my pastor told me that hiv is not something that is scary. I think that one help them to come. (interviewer: Okay). Ahaaa…, and those will continue. (interviewer: Okay).

**Interviewer:** So you, you, you started by saying that the religious leaders are part of the problem. [00:02:43] Is it that they do contribute religiously? Does the religious leaders, do they contribute to given maybe some negative information about the disease?

**Interviewee:** Yes. Yes. (interviewer: Okay). Because they always tell [00:03:00] them that if I can good the local language. [00:03:07] (interviewer: Okay). “Yadeȝ wei deȝ yatɔ ama wo” (That means someone bought the sicknesses or maybe the infection for them). As long as they think (interviewer: Okay). Even today we had the experience.

**Interviewer:** can you explain?

**Interviewee:** Okay. The man is from, uh, let me put it luckily Ashanti region. (Interviewer: Okay). He's not from this side. (Interviewer: Okay). So he came for refill (interviewer: mm-hmm). So he just came in and are attending to her, eeih, to him rather.[00:03:35] So they started and when you come try to engage you small, to know if there’s anything, maybe in, and this man was saying, “Yadeȝ wei deȝ asaase ho na ɔbi atɔn ama ooo…” [00:03:49] (Interviewer: Okay). That means, they are having land issue and the other person bought this kind of sickness or infection for him.” And I said oh, “Na agya, wote ha e, bibi hawo ana…?” (Father, as you are sitting here, is there something wrong with you?) [00:04:02] (interviewer: Okay). And here started laughing. “Se, bibi a hawo”? (I hope there’s nothing wrong with you?). (interviewer: Mm-hmm). And he said eeh “ aah..bibi a ha me ooo” (There’s nothing wrong with me…), but I have gone to four places to ask of it and they're saying the same thing “se ɔbi na ȝtɔn ama me” (Somebody bought it for me). (Interviewer: Okay, places…) like these religious leaders, visited four different religious leaders, and they are all saying the same thing that “ɔbi na ȝtɔn yadeȝ no ama” (Somebody bought the sickness for me). (interviewer: Okay, Okay). So I was just trying to put in that if these four people, (interviewer: mm-hmm) were having should I say knowledge about hiv and they're able to tell him, say, Oh, “wei ȝnyȝ homhom a deȝ” (this is not (interviewer: mm-hmm). “Yȝ ho naa dea na ho, nti kɔso na nom aduro no" ()[00:04:40] (interviewer: Okay). They'll take it from there, because they believe them, they believe them spiritually than us.

**Interviewer:** Okay, Okay. So [00:04:56] if I may come in again (interviewee: uhu…) [00:05:00] once a patient is missing that is in this case, lost to follow up (interviewee: to follow up), what are some of the challenges you face in tracing them?

**Interviewee:** Okay. Before we initiate, we, we take addresses. (interviewer: Okay). Yeah. And we, we take mobile phone numbers. (interviewer: Mm-hmm), but you know, there are in two folds. The stigma [00:05:28] attach to hiv even before you visit a client, unless he give you the consent. (interviewer: Okay) to visit him at home. (interviewer: Okay). Ahaa. Because if you visit them at home “na doctor sere o, na deȝn tea?” (Doctor came and visited you, what is the reason?). it was difficult. So they always need to give you consent before. so basically, we depend on mobile phones, the contacts to call them. (interviewer: Okay). But this our tiren, always difficult for me to get them.

[00:05:49] (Interviewer: Mm-hmm). Someone will tell you, Oh, you do verification. We always verify. Interviewer: Mm-hmm). In front of the person, because the person is in town, network is going through, you [00:06:00] can get the person, but if they get back to the villages, that one too becomes another issue. (interviewer: I see). So getting in touch that’s basic of it, but we wish we can get to them in their houses. [00:06:11] But maybe one, maybe, maybe even the transportation would be an issue. Because some places, and they don't give the give the address you always want. (interviewer: Mm-hmm). we have a case like that. They say “Joseph akoraa” (Joseph village). Always, people try to give the biggest town closer to their village to you than giving their real village. (interviewer: Okay), so this person was from “Joseph akoraa” (interviewer: Mm-hmm). So we interrogated “wo fire hi fa”?(Where do you come from?). “Me fire durowaakrom” (I come from durowaakrom). “Durowaakrom na o firee? Eehh, me fire durowaakrom” (It’s durowaakrom that you come from right? Yes , I come from durowaakrom). Agya what what “fie” (house). Not knowing this person is even coming from Joseph Akoraa. You will even get there before you get to durowaakrom. So in this case, even if you want to, [00:07:00] go there and see the person on your own, you get to durowaakrom and you never find the person.

**Interviewer:** [00:07:06] I see. Is that all the challenges with regard to tracing? The contact of such people.

Interviewee: Yeah. As I talk about the phone calls too. uh, phone calls. I think that one was the easiest way thinking it will help, but most numbers are not going through (interviewer: because of) the network challenges is one. Because where are…And these people, Oh, changing phone numbers [00:07:37] too is an issue. Because we always try to ask “yȝ ferȝ wo last time no, ȝn ba ooo…” (the last time we call you, it didn’t come through). Then the person will tell you “sȝ…maa sisa me number” (I have changed my number).

**Interviewer:** So, So what do you do to resolve that issue of patients who provide their numbers to you when they are on the drugs? Or, [00:08:00] um, in case they want to change, is there any possible means that you can do so that they can reach you or encourage them to [00:08:07] always bring back to you that I'm going to change my number because of the relevance of taking the drugs. Is there any way that you can encourage them to either always maintain on the number that they have given to you, or if they're going to change a number? They can get in touch with you. Is there anything?

Interviewer: [00:08:26] Yes. I think there’s a way, and this thing came up, I think last two years or so. (interviewer: Okay). The supply of drugs was irregular. (interviewer: Mm-hmm). So we are doing this. Always if they come, we write our contacts at the back of the appointment cards (interviewer: okay) with, the aim that before they set off from their village, we don't want them to come [00:08:48] whereby they’ll not get the drugs. (interviewer: Okay). The cost, we were just trying to do away with the cost. So we were doing this, always when they come we write our contact numbers behind the appointment cards. That before you come [00:09:00], call us before. So most people were calling (interviewer: mm-hmm). So that, that was making us to know that, okay, this person has changed the number (interviewer: mm-hmm), [00:09:10] or using a different number to call. So that's what we are basing on. So if you call, we know this number, “o aaa wo number nie?” (is this your number?), “me” (my) sister number. Then we get back to update, we will not cancelyour own. (interviewer: Okay). But we will add it to it.

**Interviewer:** Okay. That’s okay. So can you think of any situation or experiences that make it difficult for you as the health worker to find missing patients? [00:09:40] Any situation or experience that make it difficult for you to find missing patients?

**Interviewee:** Mm. Like some will be like the person contact is not going through (interviewer: Mm-hmm), and the address given too, it's not the right address, sorry to put it, (interviewer: Okay) eheee. In such situation it's always [00:10:00] difficult. With that, unless the person comes again. [00:10:04] (Interviewer: Okay). That’s where you can update. Okay.

[00:10:15] Okay. Oh last “yȝ yȝ sȝȝ yȝ ferȝ wo ooo, am ba” (we tried calling you the last time, but it didn’t come through). Babiaa wo kyȝrȝ sȝȝ wo wɔ no, yȝ baa yȝ no, ɔmo sȝȝ ɔmon nim obiaa ɔwɔ saa deen no” (where you told us that you live, when we came everybody said they don’t know anybody bearing that name). Then with that the person can give you maybe a new number. (interviewer: Okay). And maybe update the address.

**Interviewer:** Alright, alright. So, so do you see contact tracing of patients to be a matter of concern?

**Interviewee:** Yes.

**Interviewer:** Why?

**Interviewee:** Yes. we're not getting them. We want them to be on drugs always. Contact too because they're not given the right addresses, so to put, and we now reaching number on phone. [00:10:45] And in some instances, it's not that they are not on drugs, they are (interviewer: uhu). Because this is the site they were registered. (interviewer: Okay). And always, if they come and we update on the e tracker. If the person goes for drug, maybe [00:11:00] from a different facility. And it's not updated on the e tracker. Always we see such a person as default… lost to follow up. [00:11:07] (Interviewer: Okay). Which is not so. So, I don’t know if you can bridge that, because we have a lot of instances if the person go for drugs. (Interviewer: Mm-hmm). One example is, uh, a lady came. That she travelled to let me say Ashanti region, she was say…before you travel come to us and let’s talk, but she said emergency “na ȝ ba yȝ, nti me kɔ” (it was an emergency that came. So I went). They go and some are so careful about their health. So they go for the medication. [00:11:36] (interviewer: Okay). But people at the other side give them drugs, but they don't go onto the e tracker to update

**Interviewer:** people on the other side, like…

**Interviewee:** Like maybe Ashanti region. The person is from here.

**Interviewer:** Their New place where they go?

**Interviewee:** Yeah. Going for the drugs. They need to updates. Ehee. Because the person did not come to me. So on the system, you see that the person is lost to follow up. [00:11:57] Meanwhile, the person have gone for drugs [00:12:00] and taking the drugs at a different facility. But because was not updated on the system, this same person will be lost follow up. But in reality, the person taking the drugs, (interviewer: Okay). I have a.. six or four. The person went there took drugs for six good months, (interviewer: but the person is captured as lost to follow up?) the system will tell you so, because no update was in the system.

**Interviewer:** [00:12:23] Because the person hasn't come to you over and over. So I want to understand something. (interviewee: Mm-hmm). The lost to follow up nu, Is it a system that automatically captures (interviewee: Yes) the person as (interviewee: lost to follow up). Not you?

**Interviewee:** We can do it manual (interviewer: can do it manual), but when you are pulling data from the E tracker. It'll, the e tracker will tell you that because you didn't update [00:12:43] (interviewer: Okay, Okay), the person is lost to follow up, even if the person comes to you and after attending to the person, you don't go to the e tracker to should I say update (interviewer: Okay). And you put the folder aside, you don't update, the system will just tell you that the person is lost to follow up.

**Interviewer:** So on that experience, you were trying to give [00:13:00] that the person went to a different facility in the Ashanti region [00:13:03] for six good months (interviewee: going for medication), the person wasn't updated, on the e tracker. So how, how do they, when did, how, in your interaction with the person, how were they able to, you know, capture the drugs that were given to the person, or they just gave to the person to go?

Interviewee: you see [00:13:26] (interviewer: Mm-hmm). with, um, hiv, the issue that we don't want to because, we don't want them to, should I say defaults? (interviewer: Okay). Anywhere the person comes with the appointment card, you need to give drugs to the person. That was what we were doing even before the e tracker came in. So the e tracker is in now to tell you that this number of people are lost to follow up, but if you don't enter on E tracker, you never see it. [00:13:54] (interviewer: Okay). It only tells you they're lost to follow up, they're lost to follow up. But if you enter, it will tell you they're not lost to follow up. [00:14:00]. So in this situation, the person go to ehmm, Ashanti region to take the drug. Kumasi people will give the person the drugs (interviewer: Okay). But they will not maybe take the pain to go onto e tracker to update. (interviewer: Okay). Because we saw it, I saw this early, so we were doing that.

**Interviewer:** [00:14:18] How, how do you know? How do you, [00:14:26] how do you get to know that situation where the person was giving the, the drugs for the six month, continuous six month?

**Interviewee:** Unless the person comes back to you. That's when you are able identify that this person was at place for this number of period. So when we, we come we were trying to, we were trying to avoid all these things. So what we do is, we [00:14:54] always write their unique identifier number. You know the e tracker give them a unique number. (interviewer: Okay). [00:15:00] And the site too is having a number. So we were trying to write both two numbers at the back of the appointment card. (interviewer: Okay). So that anytime they comes to you, you can just update. (interviewer: Okay, okay). So if people were, if we, we agreed and were doing that, I don't think we see people getting, Maybe we'll get them but it will be few. but because we're not trying to update these things.

**Interviewer:** [00:15:28] Okay. I think that concern is properly, um. So let's move on. If you wait too long to find the patient, does it make it harder for you to bring them back like the person you presumably, or in reality, the person starts to follow up, You've waited over and over and over, and you have not been able to get the person. [00:16:00] How difficult is it for you to get such people back?

**Interviewee:** Those, maybe those who have given, uh, given us the concern that we can come to their houses. Maybe most of the time we deal with community health nurses. (Interviewer: Okay). As they're going out the communities, the person gave us the address. (interviewer: Okay). So if they go for their cwc, [00:16:22] at least you can check on this person and let’s see (interviewer: Okay). If the person is there. But the situation is always that if they’re not giving you the proper address. Then this one too become a challenge. (interviewer: A challenge). Eheee. ((interviewer: Okay. Okay).

**Interviewer:** So in case you find them, in case the person is lost to follow up and you find them, I just want to understand (interviewee: mm-hmm) in your situation (interviewee: when we get there), them uhhuh. [00:16:50] Do you, Is there, how soon is it to find such cases, like the person is assume or in reality lost to follow up and [00:17:00] you try to make contacts. (interviewee: Mm-hmm, how soon are you able to get such people?

**Interviewee:** Okay. Like the E tracker itself, as I said, it tells you people are lost to follow up depending on the period you get entered.

[00:17:12] (interviewer: Mm-hmm) . So with time we try to pull the data, (interviewer: uh, Okay). Eheee, pull the data to see. Even last koraa, we have a training, we able to do some, some g-server, it'll prompt you that, that this person is lost to follow up because the person haven't been taken drugs maybe for maybe 28 days. (Interviewer: Okay). So that will prompt you. Then we take those contact, try calling first. [00:17:36] (interviewer: Okay). Those they can get, or maybe it's, or maybe “maa tu kɔn ooo” (I have travelled ooo), others too it may not even go. So those who we cannot get them from, that's when we try to bring the community health nurses onboard. (interviewer: Okay). Oh, if go “Se wo kɔ community wei a fyȝ maame wei, wei, sȝ wo bȝ hunoa” (if you go to this community, look for this woman if will see her).

**Interviewer:** [00:17:53] So, okay, so in that case, um, you try making [00:18:00] contacts. (Interviewee: Yeah). Do you have. Um, though you've indicated that you always have internet and sorry, network challenges, but those calls you have been making, do you have a challenge maybe in getting the necessary, uh, resources, like the calls to be making that. Or is there any provision that you should, or is your own initiative that you have put in place to do those things?

**Interviewee:** [00:18:29] I think it's our own initiative. (interviewer: Okay). Uh, because data, you know, in Ghana Health Service, data comes for a purpose. (interviewer: Okay). When I give you data, they tell you that this is what you are using it for.

**Interviewer:** So, so there's no specific credit for calling?

**Interviewee:** Yes

**Interviewer:** Okay, Okay. Well what about it in general, making contact tracing, what, Do you have any challenge? [00:18:53] (interviewee: Like maybe means of?) Yes. (interviewee: The means or to buy fuel?) Yeah.

**Interviewee**: That one too is a challenge, it's a challenge, [00:19:00] that’s why basically we try to be first try to write behind community health nurses (Interviewer: uhu). Eheeee (Interviewer: okay, okay). Because If you want the system to bring you, you can get all. So we always try to take the opportunity to write behind them, (interviewer: Okay), oh, if you are to this community, please try to play up for this person for us.

**Interviewer:** [00:19:16] And do you have any challenge in them? Maybe accepting, you know, is additional role that they have to play.

**Interviewee:** Oh, they, they're willing to, they, it's not that many. (Interviewer: alright. Maybe if you're going to one community A like this, (Interviewer: Okay). you're gonna meet just one person.

**Interviewer:** So in in entirety, do you think that people who are lost full should be traced?

**Interviewee:** [00:19:40] Yes. Yes.

**Interviewer:** Okay. What about those who are maybe very sick. Such that, do you have any situation of that sort?

**Interviewee:** Yeah, we, we, we have people who cannot even work. (Interviewer: Mm-hmm) and, uh, when we started, Okay, when we started with them, they were [00:20:00] able to come in for the drugs. But later they were taken down by other sicknesses. Oh, (interviewer: that's not necessarily the…) hiv.

So with that, we asked for a relative they’ve trust. So one I know from maybe a committee B like this (Interviewer: Mm-hmm) always let the child come for it. (Interviewer: Okay, Okay, I see). So the challenge has been the viral load.... It’s another issue, uh, that means the viral load we need to go there, to take the sample. (Interviewer: Okay, okay) ahaa.

Interviewer: So these are special cases?

[00:20:32] Yes. Yeah. we don't have…

**Interviewer:** In such special cases. Definitely you have to make the effort to go. So do you have the support of either the district from the district health directorate or your facility to get in touch with such people because you have to, uh, by all means go to pick the sample from them. Because they are special cases.

[00:20:53] Or there's no, no…

Interviewee: No special support, as I said. No special support. So we always try to be smarter than the system [00:21:00] by engaging those who will go there. (interviewer: Okay). You know, these cwc at least a month they will go there, (interviewer: right) So we always try to ride behind them, “oh, o kɔ a, mon kɔ hu wei a ma yȝ” (When you go check on this person for us). But with the sample taking, that one is serious. Because maybe most… not all health workers are allowed to go into vein. (interviewer: Okay). So the community health workers may even go there, but going into vein is another issue.

[00:21:20] (Interviewer: Alright). So maybe unless we have time talk with the lab technician and maybe they give a motorbike and rush there one time.

**Interviewer:** So that's great. What are some of the challenges you face when you try to encourage patients to come back to care? That is, you realize that the person is almost getting to, uh, to default or is lost to follow up [00:21:44] totally. And you have been able to trace, like you were saying, you've gotten to the e tracker, and you've realized that the person is indicated lost to follow up. What are the challenges that you face to encourage people to come back to care in case they have all [00:22:00] actually ben lost to follow up?

**Interviewee:** If I understand, that means they were lost to follow up [00:22:05] (interviewer: Yes). But now we've be able to contact them.

**Interviewer:** Yes. And you want to now encourage them to come back to care. What are some of the challenges?

**Interviewee:** Well, maybe. Let me put it this way, Anyone who is lost to follow up apart from what we discuss. (interviewer: Okay). Which is maybe the travel, it has taken them long and it has not been updated on the e tracker. Such people if you meet them, everyone have a reason.

**Interviewer:** [00:22:28] Mm-hmm. such as…?

**Interviewee:** As I talk of the… these religious leaders. (interviewer: Okay). The person has started already. Doing well (interviewer: Oh, okay). Then later on oh “Me sɔfo sei… ȝyȝ homhom a deȝ” (My pastor said it is spiritual thing). Such person you need to take time and talk to them. Try to let them understand that no, there's no sickness now. (Interviewer: Mm-hmm). so don't think because you're healthy that’s all. The viruses are in there. I always tell them that these virus, if you're in there, we can never take them out.

[00:22:55] (Interviewer: Okay). The best we can do is just to suppress them. And [00:23:00] if you tell them that, I they will come to the understanding, that okay, this is the situation. And most of time I need to mention counseling at times, some people counseling is, they don't put it out to them in the reality of this whole thing. (Interviewer: Mm-hmm). but moment the person just comes in, [00:23:16] maybe I came in to offer self-test, for you to test me. I'm not sick.

**Interviewer:** Sorry ooo. Counselling in the site of who?

**Interviewee:** Oh, anyone who initiat, try to initiate. Because you need to counsel them before, they need to accept. (interviewer: Okay). Ehee. But if you tell the person. Maybe I'm talking with you, trying to counsel you before you start, and I'll tell you, Oh, “wo yadeȝ, wo yadeȝ”(this your sickness, this your sickness) (Interviewer: mm-hmm). But the person sitting now is not sick. But you’ve been able to identify the virus in the person. (interviewer: Okay). Now this person is not sick, so if put at the back of their mind that. Oh, “wo yadeȝ, wo yadeȝ” (this your sickness, this your sickness). I mean, ahh! If person go back home, they will sit and say ahh! But I’m not sick [00:24:00]. But you are telling me “wo yadeȝ, wo yadeȝ” (this your sickness, this your sickness). Since am not sick, there is no need for me to take this medication or come for the medications.

[00:24:03] (Interviewer: Mm-hmm). But if we make them understand that mm-hmm. , maybe you may come with some sicknesses or some sides eff… before we can test and know (Interviewer: mm-hmm). , but when we are able to identify the viruses in you. Maybe we should do away with the sicknesses itself and tell that you are have the virus now. (interviewer: Okay). You are not sick. [00:24:23] You are not at the AIDS stage (interviewer: Okay). So whatever we are doing now, it’s about you not getting to the AIDS stage (interviewer: Okay). The virus, we can't take them out. I always tell them. I know other people have the… No, no no “ɔnom saaa” (if you take it for long…), “ɔnom saaa” (if you take it for long…), the virus is there. (interviewer: Okay). The best way we can be able to know that maybe we have suppress it during viral load test.

[00:24:44] (interviewer: Okay). When you use this antibody test to do , It will show. (interviewer: Okay). Because the body have been able to identify that. So we just need to make them understand that this is two different thing. You are here now. We don't want you to get [00:25:00] here. (interviewer: Mm-hmm). That's why you go on this. (Interviewer: Okay). So all these things you're taking not that you are sick, [00:25:04] but we don't want the virus to make to make you sick.

**Interviewer:** So in situation where you have been able to encourage a person to come back. Do they? Do you what? What is the reason why those people will want to come back? The person is lost to follow up. You have identified the person, you've encourage him or her to come back to care. [00:25:26] The person has agreed to come back to care. Is there any reason that they give as to why they are coming back to care?

**Interviewee:** Yeah. As earlier... as I said, Oh, “me sɔfo sei” (my pastor said…). and some people tell say…

Interviewer: So, so sorry, but that point is well made, but I want to understand you hav... The person has agreed to come back. Why would the person agree to come back?

Interviewee: Based on the, maybe the reasons or explanation you gave about the… (interviewer: okay) condition.

[00:25:53] (interviewer: Mm-hmm). That you're taking it for life. So if I'm doing counselling, I always make you understand that you [00:26:00] are taking it for life. (interviewer: Okay) So most people I ens.. I started with, even when they travel and they are for two days or three, and they don't have medication. They’ll call you (interviewer: Mm-hmm). I have travel to this place. But my, my drugs are short. [00:26:16] It's two days now. (interviewer: Okay). Will it have any effect on me? So people who understand the counseling and they're willing and they understand the whole situation, they're willing (interviewer: Okay) when they come. But we have a, a system put in place that when you are lost to follow up like this, at times you consider distance. [00:26:35] (interviewer: Okay). So as we come, we've talked and we all agree again, we'll not start by giving you maybe multiple months dispensing. If I talk multiple months, maybe you can give you three months, six months, because the person has defaulted or lost to follow up. (interviewer: Okay). So with that, we'll talk to you and we start maybe giving you one month or two months. [00:26:57] To be able to know your adherence. [00:27:00] (interviewer: Okay). Before maybe we do the viral load and see whether it’s suppress, then can give you maybe more (interviewer: of the...) drugs.

**Interviewer:** So, [00:27:16] um, You what? What is it? Is it necessary that is there the need for us to initiate and also always encourage the contact tracing of the patients who are lost follow up?

**Interviewee:** Yes. Yes. [00:27:37] because before you initiate, you need to let them understand you're not quicking. Maybe one or two things may come up. (interviewer: Okay). Ahah, and you may not even come. But with that, don't wait. And one issue maybe just to, it just came up and some of them have issue of transportation. (interviewer: Mm-hmm. . . Mm-hmm). The person will come, should I say. Okay. They lost to follow up because they period they didn’t come within the period.

[00:27:58] But when they come [00:28:00], we try to engage them (interviewer: Mm-hmm) to know what is wrong. Oh, “me ni, man nyȝ sika ooo, ȝ saa yȝ. Me hoo yȝ. But man nyȝ sika” (I don’t have money. It got finished, I knew it, but I didn’t get money). So since, we are trying to do it this way. “Wo bȝ teaseȝ, ka obia ɔ ba weying wɔ hɔ, wo ni yȝ, na anka ɔdea berȝ wo”? (Will you agree, so that anyone of us who will be coming your community for weighing, we will give to be given to you?) But most of them decline. (interviewer: I see). Oh, we wanted to do it that way. Okay, “wo tea sea, deȝ ɔbȝ ba weying wɔ hɔ no, ɔbaa, waa duro no bȝ saa, fa wo card no ȝyȝ dȝn? Ȝ ma no,na anka ɔba,, ɔn gyia aduro no, na anka ɔmo baa ɔfan yȝ dȝn? ɔfan berȝ wo” (if you will understand, the person who will be coming to your community for weighing, if your drugs get finish, give your appointment card to them to come and take the drugs for you). [00:28:29]. But most of them, so far, they all declined (interviewer: I see. I, I see).

**Interviewer:** As a health worker. You, I know you haven't been specifically assigned to, uh, the ART clinic. You have some other responsibilities. Do those additional responsibility interfere in the effectiveness of operation of [00:29:00] your work as an ART nurse? [00:29:03] Uh, additional responsibilities that interfere in,

**Interviewee:** Okay. I would say not really. (interviewer: Okay). we don’t do clinic days. You know, some facilities they do clinic days, for all HIV related cases need to come maybe on Thursday. (interviewer: Okay). We are not doing that. (interviewer: You are not doing that…?). we're not doing that,. You know the setting we are, and if you do that, they will not even come. [00:29:28] Just imagine someone comes, (interviewer: I see), and they're together. I know you in town, you know me in town (amidst laughter), we all come. So we don't do that. (interviewer: you don't do that). You come for refill, we take care of you. You go. We also advise, when we give you let’s six months. Don't wait for it to get finished. (interviewer: Okay). Even when you are left the last month, you can come. Ahaa, that is the way we running here.

**Interviewer:** [00:29:54] I see. Okay. So in terms of contact tracing, are you always [00:30:00] giving any incentives as a contact tracer that is with regard to patient lost to follow up? Are you giving any

**Interviewee**: incentive, incentive, incentive? No (amidst laughter)

**Interviewer:** Okay. Okay. All right. Um, uh, okay. So how do you predict that a person is going, uh, as a defaulter that is, is likely to go missing? [00:30:32] How do you predict that the person is likely to be lost to follow up?

Interviewee: Okay. Um, you know, we have, uh, follow up visit dates. (interviewer: Mm-hmm). so after if the person comes today and I take care of the person (interviewer: Okay). Based on the days given, I need to estimate the next time he needs to come. (interviewer: Okay). So this date always help us to know whether the person has about maybe after months you're not seen the person. [00:30:59] (interviewer: Okay). [00:31:00] Clearly the person is close to lost to follow up and lost to follow up is lost to follow up.

**Interviewer:** Okay. So, so I want to understand, the person has come today. I have come today and I, I have been counseled and I have to be on the drugs for some time. What, how do you know that if the person I am getting closer to a point where I am likely to be lost to follow up, That's,

**Interviewee:** that’s 28 days from uhmm...

**Interviewer:** [00:31:32] Okay, so, so now the 28 days from the start of the... Interviewee: From the last day before your medication gets finished. (interviewer: Okay. Okay). 28 days if we don't get you, that means you are lost to follow up.

**Interviewer:** Okay. Okay. Alright. Is there, do you have any additional, um, information, experience, or point of view in regard to patients lost to follow up?

**Interviewee:** [00:31:56] Yeah. Uh, um, [00:32:00] I, I, I think we need to all come together and.... As already mentioned, people are on medication and they are, should I say, lost to follow up which are not too good. So the issue is I wish we should all come on board so that any time any client visit you at your facility for refill, we need to try go onto E tracker and update it.

[00:32:33] If we try to do that, most of this lost to follow up will not be the lost to follow up we try to ignore. It may be an artificial loss to follow up. But the system will tell you that this person hasn't taken drugs for this number of period. Based on that, the person is lost to follow up. But maybe in reality, the person is taking the drugs elsewhere.

[00:32:53] So I think we should all come board all sites, anytime they do refill. You should go on the e tracker [00:33:00] and update and let’s update so that this artificial lost to follow up will be very great.

**Interviewer:** Okay. Thank you much for participating in this organization. I'm most grateful.

**Participant 5**

**Interviewer:** [00:00:00] Um…, as the in charge of the facility who supervises every activity that goes on, we want to understand with regard to patients who are lost to follow up, why some individuals, who are on ART usually lost to follow up.

**Interviewee:** Oh, okay. Alight. So I think, first of all, we're happy about this study including our institution, something that improve the quality of care. [00:00:34] We think sometimes some of the clients relocate in terms of their addresses. So maybe the person previously was residing here and then move. (Interviewer: Okay). So that, that's a contributing factor. That's geographical relocation. In terms of their contacts too sometimes, the contact that they provide in terms of addresses or phone numbers becoming accessible, or when we try to call them or follow up on them, we do not reach them. (Interviewer: Okay).

And then some of them too [00:01:00] who enroll on the program sometime when they come Fridays, their market day. So you see the surge on Fridays when they come for their market. Then they, they come. (Interviewer: Okay). So if those people change their trade or some other things, or their commercial activity from Kukuom, then it become difficult to locate them.

[00:01:18] And some of them also do not have addresses at all or phone numbers. (Interviewer: Okay). Uh, to, to get in contact with them. (Interviewer: Okay), so. I think these are some of the things we…

**Interviewer:** Okay. With regard to the, um, addresses or location given wrong location, um, to, or addresses to the, uh, facility in order to ensure that, uh, you, uh, Uh, effective contact tracing and or follow up. [00:01:48] What are the possible means of addressing that?

**Interviewee:** Yeah, so what the unit is putting together is that when they register and they give them [00:02:00] the contact, now you've provided them a phone. (Interviewer: Okay). So instantly you can call on the line (Interviewer: Okay) and make sure that it's an active number. (Interviewer: Okay). Also, when they give you the address, [00:02:10] let's say if the person say, I stay behind so so and so mm-hmm. , they can give them a proxy now. Now the challenge is that because of the societal stigma associated with the condition (interviewer: Mm-hmm), people do not want to be relatable. (Interviewer: Okay). So that you can be able to know that he stays behind so so and so landmark in terms for following up. [00:02:30] So, (Interviewer: Okay). In terms of the phone numbers, that's what we intend to do call on the line, make sure that it’s active. (Interviewer: Okay).

**Interviewer:** Okay. So, um, as the med soup of the facility, um, what are some of the experiences or situations in which you encounter with regard to difficulty in, uh, getting in touch with (Interviewee phone vibrated) some of these people, like those who [00:03:00] are on ART or diagnosed of the disease and are put on the drugs?

**Interviewee:** [00:03:06] Okay. I think some of the, the counseling needs to be quite detailed enough. For some people who, let's say default treatment, let me use example of those that we are a… defaulters we are able to find. (Interviewer: Okay). Those who default treatment sometimes they visit other places, including herbal people and religious bodies or herbalists, traditionalist, and they are given them some Concoction and tell them that they've cure them of the disease.

[00:03:37] Some actually come back and want to retest. (Interviewer: Okay). You see? Told they went somewhere, they've been told that the disease, they've been given something or they've been prayed for, so the disease has been vanished or cured. (Interviewer: Okay). Now they retest and the thing is still there. (interviewer: I see). So I believe we need to do more with the counseling.

[00:03:55] (interviewer: Okay). Secondly, I think, uh, there's also been [00:04:00] some reported instances of perceived stigma. Mm-hmm. even within the community or people thinking that maybe if go to healthcare workers. So we, we working hard to secure our folders in terms of the, the, the people living with hiv..,, (Interviewer: Okay) folders. Ahaa. And then also doing more training for the staff in terms of, uh, patient confidentiality and then minimizing or preventing stigma associated persons leaving with hiv.

**Interviewer:** [00:04:32] Okay. In terms of the securing of patients folders, I want to understand, further, how do you as a facility ensure that?

**Interviewee:** So before there was no lockers for their folders. (Interviewer: Okay). It was on shelves. (Interviewer: Okay). So now I think we've provided some lockers to be able to keep (Interviewer: specifically for the a..?) Yes. For, ART.

**Interviewer:** Okay. That's great. Okay. [00:04:58]. That's fine.

**Interviewee:** Yeah.

**Interviewer:** So, [00:05:00] um, is there the need for contact tracing that is with regard to patients on art?

**Interviewee:** Oh, yes. It's, it's very important. We believe that one, it enhances the adherence to treatment. (Interviewer: Okay). And then also you are able to get the patient lead experiences if you do contact tracing. So now you know the region through JSI was doing a contact tracing enhancing and linkages to care.

[00:05:28] (interviewer: Mm-hmm). So one of the challenges initially was funding. (Interviewer: Okay). Now we secured funding that went into purposely for contact tracing. So we provided credit and data. (Interviewer: Okay) to the art team. And now they're able to call the people frequently (Interviewer: Okay) to find out how they're doing and how, I mean, they, they're taking their medication and they have side effects or other things too, they are also coached, (Interviewer: Okay).

**Interviewer:** [00:05:59] Um, [00:06:00] further I would want to, um, find out how convenient it is, with regard to the hours that people or, uh, patients on ART come to the facility for their drugs, do you have any experience as to how they express, um, their experiences with regard to the um, the convenience of the clinic hours.

Interviewee: Well, for, for now [00:06:28] I, I don't have information on it, not being, but we run throughout the whole week, Monday to Friday. (Interviewer: Okay). So, but I think maybe we, the weekend that the think ART clinic is not operating on the weekend. (Interviewer: Okay). So, Like if any inconvenience, I think that might be the challenge. So, (Interviewer: Okay) well we can look at a survey, and ask people if the weekends would be more convenient, we can reinforce our staff and ensure that (Interviewer: Okay). [00:06:56] weekends too do operate, but for now it doesn't operate on the weekends.

**Interviewer:** [00:07:00] Okay. So briefly, what, how do you describe the characteristics of the clinic?

**Interviewee:** Well, uh, so characteristics as in…?

**Interviewer:** Like, maybe where it is situated. (interviewee: Oh, okay). Um, like is, it’s accessibility with regard to other people coming in when clients coming in?

**Interviewee:** [00:07:19] Yeah. Okay. So I think, I mean, that's much clearer. Uh, in fact the, there's. The place where the ART clinic is in itself is not conducive structurally. (Interviewer: Okay). So there's a infrastructural challenge, there’s infrastructural challenge (Interviewer: Okay). For the infrastructural challenge. So, um, that's limit the accessibility to care. (Interviewer: Okay). Um, also, it is also a shared space with mental health unit.

[00:07:46] (Interviewer: Okay). Uhhuh. So I think that also in itself is a challenge and it's a challenge. And within the same complex as maternity. (interviewer: Mm-hmm). Uh, so these three major structural situational characteristics [00:08:00] affects accessibility. (interviewer: Mm-hmm). And then also it affects the patient's confidentiality. (interviewer: Okay). Because I mean it’s within the same thing, that also run a mental health clinic.

[00:08:12] (interviewer: Mm-hmm). And all of that. Yeah. And it's also a very small, limited space. So other logistics, like their furniture is also challenge. (interviewer: Okay). And all of that.

**Interviewer:** So , So how do we address that?

**Interviewee:** Yeah, so now uh, we as hospitals we identified that as a big challenge and (interviewer: Okay). You know, they're doing as expansion works. (interviewer: Okay). So the maternity new block is ongoing.

[00:08:37] So we hope that if we finish and relocate the maternity unit now to the new maternity, we free up some space (interviewer: Okay) for mental health and uh, ART, yeah. And then also, yeah. Um, yeah. So those are the possible ways we are looking at. We also speaking with other stakeholders and partners, if we can have a new construction of [00:09:00] an ART space

**Interviewer**: Specifically?

**Interviewee** [00:09:01] Yes. Yes. For ART, and other health promotion activities. Because, now, in fact, one other things too, we are looking at, because of the limitation to space. Is to send the healthcare to the doorstep, to the people (interviewer: mm-hmm). , uh, in the interim that looks more feasible. Because construction will take some time. (interviewer: Mm-hmm). But definitely since you need a center of care in the facility in the long term, that's what we are advocating for so that they can be purposely an ART.

**Interviewee:** [00:09:27] Okay. What we also realize in regional centers or regional hospital that have designated ART centers is that sometimes it also increases the stigma. Because it's designated, everybody knows that (Interviewer: Okay) whoever is entering here is most likely of going with this particular condition. So other places too have done the integrated care.

[00:09:49] (interviewer: Mm-hmm). so that other people too can walk in with other conditions. (interviewer: Ok). Let's say like the wellness clinic. (Interviewer: Yeah). So anybody know that I can walk in, like this is a wellness clinic. I [00:10:00] don't need to be sick. I can walk in and go have counseling session. But within that there are different rooms. So maybe somebody is going to, maybe nutrition room, another person is going to mental health.

[00:10:09] So in the new block if, if we secure any funding, that would be the purpose. It will be multi purpose center seven, ART, health promotion, wellness clinic, so that it also move that barrier of stigma or if see, Oh, this is an ART or hiv provider center. (interviewer: Yeah). It, it, it, it, it's also kind of, uh, increases the stigma.

[00:10:33] Because everybody knows that entry here, this particular, service you are going to assess. So I mean, that's what we are doing.

**Interviewer:** Okay. that’s great. I see. So as the med sup of the facility, what are some of the job responsibilities that compete with other staffs with regard to the care of hiv? That's those who specifically are on art. [00:10:56] Is there any other competing job responsibility that [00:11:00] interferes with their, uh, conduct of contact tracing or the, um, follow up? (Interviewee: Yeah) to patients who are on art.

**Interviewee:** Okay. You know (amidst laughter), healthcare, even if you're assigned to a particular role, (Interviewer: okay), because of the limited human resources, you still find time to do other things (Interviewer: okay).

[00:11:18] So less the pharmacist is also at ART, he has to oversee drug and regulated issues in the general clinic. So we don't have designated pharmacist purposely for ART clinic. (Interviewer: okay). For, for, we have assigned a nurse, purposely for art. (interviewer: Mm-hmm). . But this nurse also does other duties including, uh, I mean, supporting the claim processing and other things. [00:11:38] (Interviewer: okay). Yeah. And then also, uh, well, So those are some of the other general duties that nurses within this unit would perform. Trainings and other things too would, although it's important to upgrade their skills, it's also one way or the other distract their (Interviewer: okay) schedule. (Interviewer: Okay). But [00:12:00] what we do is that, uh, we also able to get other train staff to augment when their workload increases.

[00:12:06] Mm-hmm. So that's what I wanted to, (Interviewer: Okay).

**Interviewer:** So, um, do you see as the manager of the facility, the need for people, uh, that’s the ART nurses or people who are designated purposely to take care of people who are on the drugs, Do you see any need of maybe getting any incentives for such people that's health workers?

**Interviewee:** [00:12:33] Oh yes. I think it's important. At our level, we provide them support in terms of their core call credit, they come for in charges meeting and we support them with some funding, but that is limited. (Interviewer: Okay). The amount is not… So we hope that, (Interviewer: Okay), if we are able to mobilize additional resources, definitely we need to motivate the staff very well.

[00:12:56] It's not only in terms of money, we support their training. [00:13:00] (Interviewer: Okay). And we able to pay for their trainings or support their transportation and other things to go and enhance their knowledge to come and then also serve the people. But if beyond all of that, there are other monetary benefits that we'll be able to assign, well, will gladly welcome it.

**Interviewer:** [00:13:19] (Interviewer: Okay), so with I one, understand. With regard to patients who are likely to be lost to follow up, how do you always ensure or identify that this particular person is likely to be lost to follow up? (interviewee: Yeah) If the person is on the ART.

**Interviewee:** Okay, so. Uh, first of all, we will start with the registration and then counseling, even before enrollment to care. [00:13:46] (Interviewer: Okay). When we, we, during the counseling process, um, I mean, you, you'll be able to potentially detect people who, in terms of acceptance of their diagnosis becomes a challenge. You, you [00:14:00] may have to do risk stratification to know that this a high risk profile patient who may default, (Interviewer: Okay). In terms of the patient support system, their family support system too should be....

[00:14:09] So now what we plan to do is that when we enroll them, we are working with community health nurses who do home visits (Interviewer: Okay). To also assign, so that as part of their specialized home visits, they can visit them of these clients and check on how they are doing. (Interviewer: Okay). Uh, so when we do that, uh, we believe that, uh, at least it would, it would enhance their communication with healthcare workers.

[00:14:35] O(Interviewer: Okay). Uh, in the counseling sessions, too we want the patient to also have some level of commitment or responsibility on their path to enrollment to care. (Interviewer: Okay). But when we also let them know that they also have a role to play (interviewer: mm-hmm). In terms of you know, taking their medication (interviewer: Mm-hmm), they're having any, uh, adverse effect or event they, they they speak out to and all of [00:15:00] that. [00:15:00] Uh, I mean, that, that would help.

**Interviewer:** All right. In summary, what do you say with regard to people who are, uh, who are likely to be lost to follow up, who lo who are lost to follow up? In summary, what do we say as a, the, the, the medical superintendent of this facility?

Interviewee: Yeah, so, um, I think, I mean, clients to follow up, patient receiving ART services lost to follow up is a big challenge. [00:15:33] One, it's, it's, it increases resistance (Interviewer: Okay) to the antiretroviral medications. (Interviewer: Okay). Um, it, it's cost to the health system as well. Interviewer: Mm-hmm). um, it also puts the community at risk because of transmission. I (interviewer: see). Yeah. So, uh, it's a big challenge and we, we, we are committed to putting that best we can (Interviewer: Okay) to ensure that we find the cases we put them on treatment, [00:16:00] they stay on treatment.

For any reason or the other is a defaulter (Interviewer: Okay), we will do our best to try and follow them up. But if it become that, becoming increasingly difficult to follow, which we term them as being lost follow up. (Interviewer: Okay). That is not good enough, and we will do our very best to, I mean, trace them and link them back to care (Interviewer: Okay).

So, but doing all this also comes with other challenges at the unit. [00:16:26] In terms of limited infrastructure, the human resource. (Interviewer: Okay). In terms of other resources, like funding (interviewer: Mm-hmm) for the home, the home visits, the contact tracing, transportation, the call credit, and all of that.

So all partners who are into HIV service, pro provision (interviewer: Mm-hmm). If, I mean, they can, I mean, give more attention to, (Interviewer: Okay) [00:16:51] contact tracing like we did during the covid time, which we, we know that (Interviewer: Okay) it helps very well (Interviewer: Okay, okay). I think it'll help to improve the, [00:17:00] the quality of care for our clients (Interviewer: Okay).
