# Supplementary material for: Factors influencing patients on antiretroviral therapy loss to follow up: A qualitative analysis of healthcare workers perspective
Source: PLoS One. 2024 Jun 13;19(6):e0304592. doi: 10.1371/journal.pone.0304592 (PMC11175404; doi:10.1371/journal.pone.0304592)
Supplement: Attachment — Submitted filename: Response to Reviewers.docx [file pone.0304592.s003.docx]

Robert Kogi

P.O. Box 22

Kukuom

The Editor

PLOS ONE Journal

Dear Editor,

**Response to Reviewers**

I am happy to bring to your notice that, I have responded to all the comments made on our manuscript. The responses to the individual comments are appropriately reported in the table below for your consideration.

Thank you.

Robert Kogi.

| **Comments** | **Responses** |
| --- | --- |
| **Academic Editor’s comments** | |
| Data availability: Please indicate that data is submitted under "additional documents" | I have added that “the data is submitted under additional documents” in the submission system |
| Material and methods – please define “retention” and “LTFU" in the context of this study, using reliable references for the definitions. | I have defined retention on care as “patients who, in the 12 months preceding to this study, made at least 4 appointments, with at least 1 visit per quarter (14), and patients who are alive and receiving highly active antiretroviral therapy by the time of conducting this study (15).” And LTFU as “patient (s) not being on ART for more than one month (16)” with their appropriate references in page 6. |
| Sampling method and sample size: Purposive sampling used - what criteria used in the selection of the participants (both patients and healthcare providers) | I have stated the criterion for selecting the respondents (Healthcare providers) as “Purposive sampling was used to identify the health workers based on their knowledge and role in management of HIV patients” in page 6. However, that of the patients was left out because of the expunction of the patients from the entire work as suggested by the Reviewer #2 |
| Data analysis – please indicate what coding approach was used (deductive vs inductive). | I have stated that “We used both deductive and inductive coding approaches in this study. Deductive coding was done with predefined codes or categories being applied based on existing theory or literature (in the basic and organizing themes), while elements of inductive coding was done with themes and patterns allowed to emerge directly from the data (labelled codes) in Table 1.” In page 7 |
| Discussion – line 555: what does “Major challenges in carrying out lost to follow up” mean? Please adjust the phrase. | This was addressed as “Challenges facing conduct of patient loss to follow-up exercise” |
| We note that the grant information you provided in the ‘Funding Information’ and ‘Financial Disclosure’ sections do not match. | This has been corrected and added in the cover letter as “Funding for this project was provided by Access and Delivery Partnership (ADP), supported by the Government of Japan and led by the United Nations Development Programme, in collaboration with the World Health Organization’s Special Programme for Research and Training in Tropical Diseases (TDR) and PATH (Grant number: UGSPH/CIRT-M/003).” |
| Before we proceed with your manuscript, please also provide non-author contact information (phone/email/hyperlink) for a data access committee, ethics committee, or other institutional body to which data requests may be sent. | The data for this study is deposited in the DRYAD Data repository platform with DOI: 10.5061/dryad.ngf1vhj25, and can also be requested from non-author (Seraphine Dzah) through [banbesera1@gmail.com](mailto:banbesera1@gmail.com) |
| **Reviewer #1** | |
| I do have some concerns about language (grammar, etc.), but these are minor and revisions have been recommended. | The manuscript has been revised entirely to correct minor identified grammar as indicated throughout the content |
| **Reviewer #2** | |
| First, the study participants is a mix of healthcare workers and patients on antiretroviral therapy (ART). These two groups have different perspective on factors that influence loss to follow up. Also, considering the non-probability sampling technique that has a higher representation of healthcare workers. The paper will be better focussed if the perspectives of one group either the healthcare workers or patients on ART is presented in this qualitative research. Secondly, the title should be modified to reflect the study participants see suggestion: "Factors influencing patients on antiretroviral therapy loss to follow up: a qualitative analysis of healthcare workers perspective." | The content of the manuscript has been appropriately revised to focus on healthcare providers only.  The title has also been modified as follows: “**Factors influencing patients on antiretroviral therapy loss to follow up: A qualitative analysis of healthcare workers perspective”** |
