# Supplementary material for: Factors influencing patients on antiretroviral therapy loss to follow up: A qualitative analysis of healthcare workers perspective
Source: PLoS One. 2024 Jun 13;19(6):e0304592. doi: 10.1371/journal.pone.0304592 (PMC11175404; doi:10.1371/journal.pone.0304592)
Supplement: Attachment — Submitted filename: Response to Reviewers.docx [file pone.0304592.s004.docx]

Robert Kogi

P.O. Box 22, Kukuom

08/05/2024

The Editor

PLOS ONE Journal

Dear Editor,

**Response to Reviewers**

I would like to bring to your notice that, I have responded to all the comments made on our manuscript based on the reviewer #2 comments. The responses to the individual comments are appropriately reported in the table below for your consideration.

Thank you.

Robert Kogi.

| **Comments** | **Responses** |
| --- | --- |
| **Reviewer #2** | |
| 1. Sampling method and sample size are not described under the methods and materials section. Kindly revise with a brief description of the sampling method and sample size of participants interviewed. | This section of the manuscript has been revised as follows: “The total sample used in this study was seven (7). This included one doctor, one physician assistant, three ART clinic staff, two community health workers (who were experienced in seeking patients in the community).  Purposive sampling was used to identify the health workers based on their knowledge and role in management of HIV patients.” |
| 2. Lines 107 - 109 reads "Eligible HIV patients on ART, as well as those on ART but lost to follow-up, were contacted via phone calls by ART nurses to explain the study, and those who accepted were scheduled for face-to-face structured interviews." Kindly delete or explain why patients are being interviewed. | The sentence "Eligible HIV patients on ART, as well as those on ART but lost to follow-up, were contacted via phone calls by ART nurses to explain the study, and those who accepted were scheduled for face-to-face structured interviews." Has been deleted from Lines 107-109. |
| 3. Most of lines 105 - 126 describes the study procedure. Kindly consider "Study Procedure" as a more appropriate subheading. | A subheading in Line 110 has been added as "Study Procedure" |
| The result section should present sociodemographic and occupational characteristics of the healthcare workers interviewed as participants in this study. | Sociodemographic and occupational characteristics of the healthcare workers have been added in Lines 145-150 |
